# The nucleosome position-encoding WW/SS sequence pattern is depleted in mammalian genes relative to other eukaryotes

**DOI:** 10.1093/nar/gkz544

**Published:** 2019-06-19

**Authors:** Gregory M Wright, Feng Cui

**Affiliations:** Thomas H. Gosnell School of Life Sciences, Rochester Institute of Technology, 85 Lomb Memorial Drive, Rochester, NY 14623, USA

## Abstract

Nucleosomal DNA sequences generally follow a well-known pattern with ∼10-bp periodic WW (where W is A or T) dinucleotides that oscillate in phase with each other and out of phase with SS (where S is G or C) dinucleotides. However, nucleosomes with other DNA patterns have not been systematically analyzed. Here, we focus on an opposite pattern, namely anti-WW/SS pattern, in which WW dinucleotides preferentially occur at DNA sites that bend into major grooves and SS (where S is G or C) dinucleotides are often found at sites that bend into minor grooves. Nucleosomes with the anti-WW/SS pattern are widespread and exhibit a species- and context-specific distribution in eukaryotic genomes. Unlike non-mammals (yeast, nematode and fly), there is a positive correlation between the enrichment of anti-WW/SS nucleosomes and RNA Pol II transcriptional levels in mammals (mouse and human). Interestingly, such enrichment is not due to underlying DNA sequence. In addition, chromatin remodeling complexes have an impact on the abundance but not on the distribution of anti-WW/SS nucleosomes in yeast. Our data reveal distinct roles of *cis*- and *trans*-acting factors in the rotational positioning of nucleosomes between non-mammals and mammals. Implications of the anti-WW/SS sequence pattern for RNA Pol II transcription are discussed.

## INTRODUCTION

The eukaryotic genome is organized into chromatin in which the access of protein to DNA is tightly regulated to facilitate genomic functions such as transcription, replication and repair. The basic repeating unit of chromatin is the nucleosome core particle (NCP) that consists of a histone octamer, around which 147 base pairs (bp) of DNA is wrapped in ∼1.7 turns (1). One of the most intriguing questions in chromosome biology is to understand where nucleosomes are positioned and how specific positioning of nucleosomes affects transcription factor (TF) binding and gene expression in the context of chromatin. Experimentally, genome-wide nucleosome positions are usually mapped by two types of methods. The first type involves treating chromatin with high concentrations of micrococcal nuclease (MNase) followed by massively parallel sequencing (denoted as MNase-Seq). However, MNase has strong sequence preferences: it cuts predominantly within AT-rich sequences in free DNA (2,3) and in linker DNA between nucleosomes (4,5). This sequence specificity can be overcome by a method based on site-directed hydroxyl radicals (denoted as chemical method) (6). The chemical method allows the mapping of nucleosome dyad positions at a single base-pair resolution (7–9) and recently was applied to the yeast (10–12) and mouse (13) genomes.

Historically, nucleosome positioning is usually characterized by two parameters: rotational positioning, defined by the side of the DNA helix that faces the histones, and translational positioning, defined by the nucleosome midpoint (or dyad) with regard to the DNA sequence (14). Several *cis* and *trans* determinants of the translational positioning of nucleosomes have been reviewed in literature (15).

With regard to the rotational positioning, it is well established that DNA sequence is a critical determinant (16) and various sequence patterns have been proposed (17–26). One of the most well-known patterns is the WW/SS pattern (where W is A or T and S is G or C), which was first described by Travers and colleagues (27). Specifically, WW dinucleotides tend to occur at the sites where nucleosomal DNA bends into minor grooves (i.e. minor-groove bending sites or minor-GBS) facing toward the histone core, while SS dinucleotides are often positioned at the sites where nucleosomal DNA bends into major grooves (i.e. major-groove bending sites or major-GBS) facing toward the histone core. This pattern has been successfully used to predict the rotational positioning of nucleosomes (28). An anti-WW/SS pattern was described in which WW runs inverse to SS, and it was found that promoter nucleosomes in yeast favor this pattern (29). That is, the number of nucleosomes following the anti-WW/SS pattern exceeds or equals to the number of nucleosomes following the well-known WW/SS pattern in yeast promoters. Note that other sequence patterns are also described (29), including RR/YY and anti-RR/YY patterns (where R is A or G and Y is C or T), and these patterns are beyond the scope of the present study.

Structurally, highly conserved ‘sprocket’ arginine residues (30,31) insert into the minor-GBS (32). Short poly(dA:dT) stretches help to narrow DNA minor grooves, potentially enhancing electrostatic interactions between DNA phosphate backbone and ‘sprocket’ arginine residues (33–35). These arginine-DNA contacts that occur every ∼10 bp within NCP provides the structural basis for the rotational positioning of nucleosomes (1,33–35). In this light, anti-WW/SS nucleosomes seem to have unfavorable DNA-histone interactions, representing a relatively unstable structure. However, many questions of how anti-WW/SS nucleosomes are distributed across eukaryotic genomes and what factors influence their abundance remain poorly understood.

Here, we have quantified nucleosomes with or without the WW/SS sequence pattern, and systemically analyzed the fraction and the distribution of anti-WW/SS nucleosomes across five eukaryotic genomes under different cellular and growth conditions. We found that anti-WW/SS nucleosomes are widespread in the genomes regardless of the mapping methods and distributed in a species- and context-specific manner. In non-mammals including yeast, anti-WW/SS nucleosomes are not enriched in promoters. However, in mammals (e.g. mice and humans), this type of nucleosomes is enriched in promoter and genic regions but not in repetitive DNA elements. The enrichment of anti-WW/SS nucleosomes is positively correlated with RNA Pol II transcriptional levels, but negatively correlated with the presence of the periodic WW (or SS) pattern. Moreover, we found that chromatin remodelers have an impact on the number but not the distribution of anti-WW/SS nucleosomes in yeast. Our results indicate that *cis-* and *trans*-acting factors play distinct roles in controlling the rotational positioning of nucleosomes between non-mammals and mammals.

## MATERIALS AND METHODS

### Definition of Minor- and major-groove bending sites in a nucleosomal DNA fragment

Minor- and major-groove bending sites (GBS) in a 147-bp nucleosomal DNA fragment were defined previously (36). Briefly, minor-GBS are located at the superhelical locations (SHL) ±0.5, ±1.5, …, ±6.5, while major-GBS are located at SHL ±1, ±2, …, ±6. Each site is 3 or 4 bp in length. In total, there are 14 minor-GBS and 12 major-GBS along a 147-bp NCP fragment. Note that the minor-GBS at SHL ±0.5 (shown in gray in Supplementary Figure S1) are not included for analysis because out-of-phase WW/SS peaks were observed at these two sites (27). As a result, 12 minor-GBS covering 48 bp, and 12 major-GBS covering 44 bp are used in our analysis (Supplementary Table S1). Since each minor-GBS (4 bp in length) holds three unique dinucleotides, the maximum number of dinucleotides in 12 minor-GBS is 36 ( = 3 × 6 × 2). Similarly, the maximum number of dinucleotides in 12 major-GBS is 32 ( = (3 × 4 + 2 × 2) × 2). To account for this difference, we assign a coefficient of 1.125 ( = 36/32) to the number of WW or SS dinucleotides occurring at major-GBS (Table 1).

**Table 1.** Classification of nucleosomal DNA sequence patterns

Sequence Type	WW	SS
Type 1	minor WW ≥ Major WW * coef^†^	minor SS ≤ Major SS * coef
Type 2	minor WW ≥ Major WW * coef	minor SS > Major SS * coef
Type 3	minor WW < Major WW * coef	minor SS ≤ Major SS * coef
Type 4	minor WW < Major WW * coef	minor SS > Major SS * coef

^†^coef: the coefficient is a constant of 1.125 ( =36/32). It refers to the ratio between 36 dinucleotide positions in minor-groove bending sites and 32 dinucleotide positions in major-groove bending sites (see Supplementary Table S1).

### Classification of nucleosomes with different NPS patterns

**Figure 1.**
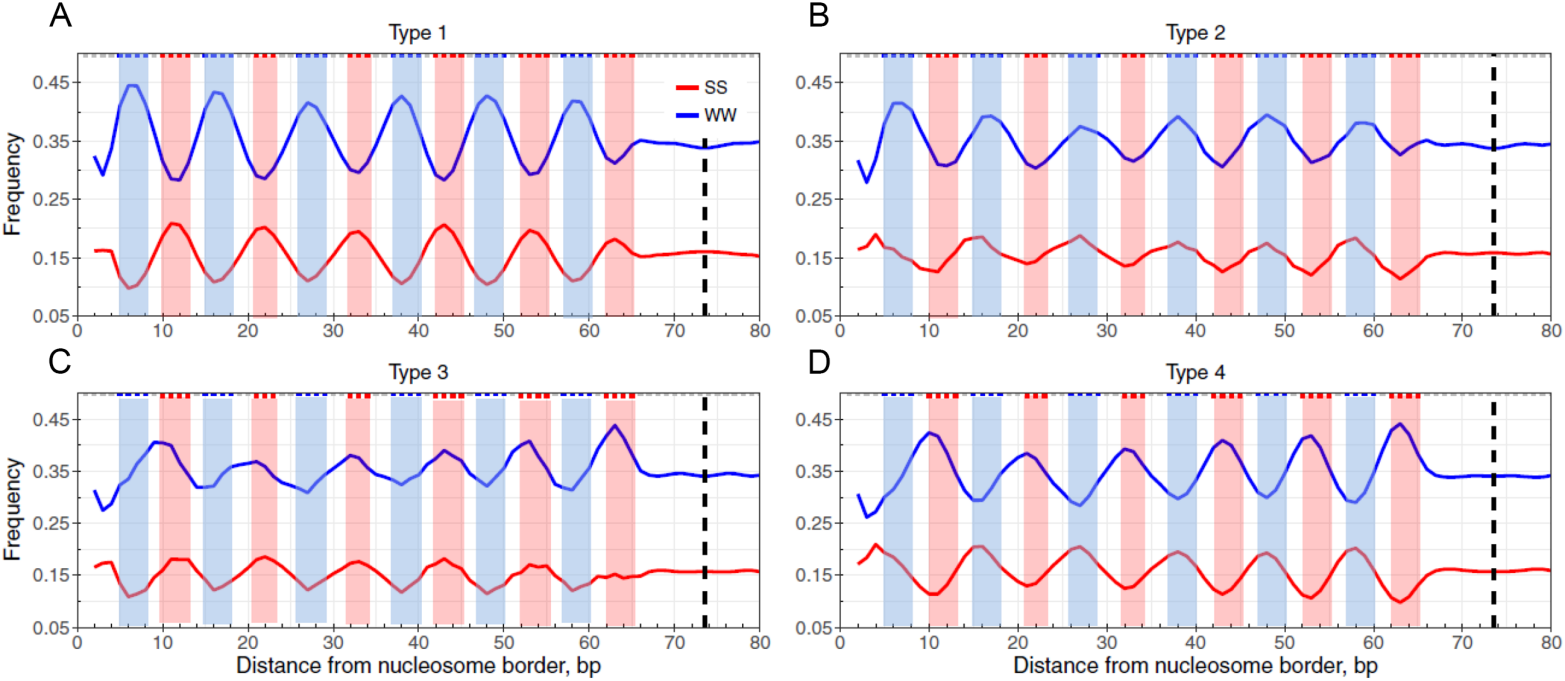
Four sequence patterns of nucleosomal DNA in yeast. Shown are frequencies of the combined AA, TT, AT and TA dinucleotides (denoted as WW, shown in blue) and GG, CC, GC and CG dinucleotides (denoted as SS, shown in red) at each nucleosomal position, which were ‘symmetrized’ with respect to the dyad (dashed lines). Three base-pair running averages of the WW and SS frequencies were calculated and plotted: Type 1 (**A**), Type 2 (**B**), Type 3 (**C**) and Type 4 (**D**). The blue and red shade areas cover the range of minor- and major-GBS, respectively (see ‘Materials and Methods’ section). The Type 1 pattern refers to the WW/SS pattern, whereas the Type 4 pattern refers to the anti-WW/SS pattern.

Nucleosomal DNA sequences are divided into four types based on relative abundance of WW and SS dinucleotides in minor- and major-GBS (Table 1). Type 1 nucleosomes have the well-known WW/SS pattern (i.e. WW are more abundant in minor-GBS than in major-GBS, whereas SS are more abundant in major-GBS than in minor-GBS). Specifically, for a given 147-bp NCP fragment, if the total number of WW dinucleotides in the 12 minor-GBS is greater than its counterpart in the 12 major-GBS, and the total number of SS dinucleotides in the 12 major-GBS is greater than its counterpart in the 12 minor-GBS, this fragment is classified as Type 1 nucleosomal DNA (Table 1 and Figure 1A).

Type 4 nucleosomes are characterized with an anti-WW/SS pattern. That is, if a 147-bp NCP fragment has more WW dinucleotides in 12 major-GBS than in minor-GBS, and has more SS dinucleotides in minor-GBS than in major-GBS, this fragment is classified as Type 4 nucleosomal DNA (Table 1 and Figure 1D). Type 2 and 3 nucleosomes have ‘mixed’ patterns, in which both WW and SS are abundant in minor-GBS (Type 2, Figure 1B) or in major-GBS (Type 3, Figure 1C). Specifically, a Type-2 NCP fragment has more WW and SS dinucleotides in minor-GBS than in major-GBS, while a Type-3 NCP fragment has more WW and SS dinucleotides in major-GBS than in minor-GBS. For a given genomic region, the difference between Type 1 and Type 4 nucleosomes in percentage (%) is denoted as ΔNPS. That is, ΔNPS = Type 1 (%) – Type 4 (%).

### High-resolution nucleosome datasets in yeast and higher eukaryotes

A total of 18 high-resolution nucleosomal DNA datasets from various eukaryotes were used in this study (Supplementary Table S2), in which nucleosome positions were mapped by the MNase-Seq method or the chemical method. Two datasets, one from yeast (10) and the other from mouse (13), were generated by the chemical method, and the dyad positions were precisely mapped at the single base-pair resolution. These positions were obtained from the published papers (10,13). Other 16 datasets were produced by paired-end MNase-Seq. Because this sequencing technique generates short reads on both ends of a nucleosomal DNA fragment, after mapping them to the reference genome, the length of the fragment can be precisely determined. Only the fragments of 147 bp in length were analyzed in this study (Supplementary Tables S3 and S4). This is because that in this case, minor- and major-GBS can be determined in an unambiguous manner based on a high-resolution nucleosome crystal structure with a 147-bp DNA fragment (36, also see Supplementary Figure S1).

Paired-end nucleosome sequence data from yeast include wild-type (WT) cells YPE458 (37), and MCY3647 (38) and mutant (MT) cells (37,38) including single mutants (*isw1Δ, isw2Δ, chd1Δ, rsc8Δ*), double mutants (*isw1Δ isw2Δ, isw1Δ chd1Δ, isw2Δ chd1Δ*), and triple mutants (*isw1Δ isw2Δ chd1Δ*). The paired reads were aligned to the *Saccharomyces cerevisiae* genome using ELAND (Illumina). Only the reads uniquely aligned to the genome with no mismatch were selected. Note that all yeast strains (WT or mutants) were grown to mid-log phase in synthetic complete (SC) medium. All of the WT strains (YPE458 and MCY3647) should be equivalent for most purposes, since they grew in the same SC medium.

Paired-end MNase-seq data were also taken from *Caenorhabditis elegans* embryos and sperms (39), *Drosophila* S2 cells (40,41), mouse embryonic stem cells (mESC) (13) and human lymphoblastoid cell lines 18508 and 19238 (42). BAM files were either downloaded from NCBI GEO database (37,38,42) or obtained by mapping raw reads to the corresponding genomes (13,39–41) using the default setting of Bowtie 2 (43), i.e. –sensitive, -I 0, -X 500.

### Genome-wide gene expression (RNA-seq) data in yeast and higher eukaryotes

The RPKM (Reads Per Kilobase of transcript per Million) values in RNA-seq data of *C. elegans* embryos (GSM1652723) and sperms (GSM3188165), Drosophila S2 cells (GSM410195), mouse mESCs (GSM2183915) and human lymphoblastoids (GSE121926) were used to divide genes into four groups based on transcriptional frequencies (Supplementary Table S5).

The yeast RNA-seq data for the SC condition were used for analysis (44). Raw reads were mapped to the yeast genome by the default setting of Bowtie (43). The RPKM values of the yeast data were calculated following the same method as the human RNA-seq data (45).

### Repeat elements in the mammalian genomes

Mouse (mm10) and human (hg19) repetitive region positions were downloaded from the UCSC Genome Browser. The repeat elements were identified using RepeatMasker (v3.2.7) and Repbase Update (9.11). The major types of repeat elements were selected for analysis, including short interspersed nuclear elements (SINE) (Alu, MIR), long interspersed nuclear elements (LINE) (CR1, L1, L2 and RTE), long terminal repeats (LTR) (ERV1, ERVK, ERVL and Gypsy), Simple Repeat ((TG)_n_, (TCG)_n_, (CACTC)_n_, (GAGTG)_n_ and (TATATG)_n_). The remaining repeat elements were included into the ‘Other’ category including MuDR, PiggyBac, TcMar-Mariner, hAT-Charlie. Only the elements with >150 bp in length (i.e. approximately the size of one nucleosome) were selected for analysis.

Since human nucleosomes were mapped to the genome assembly hg18 (42), the UCSC LiftOver utility was used along with the hg18-hg19 chain file to convert the hg19 repeat elements to their corresponding positions in human genome assembly hg18. Nucleosomes overlapped with these repeat families were summarized in Supplementary Table S10.

### Distance auto- and cross-correlation function

The distance auto-correlation (DAC) and distance cross-correlation (DCC) functions have been discussed previously (46,47). In the present study, DAC function was used to calculate the correlation between WW or SS dinucleotides. That is, if a WW dinucleotide (AA, TT, AT or TA) is separated from another WW dinucleotide by a distance *d*, one occurrence is counted for that distance in the DAC function. For the DCC function, we counted how many times a WW dinucleotide is separated from a SS dinucleotide (GG, CC, GC or CG) by a distance *d*, where *d* is between 1 and 150 bp.

## RESULTS

### Anti-WW/SS nucleosomes are widespread in eukaryotes

NCP fragments of 147 bp in length were divided into four sequence patterns, Type 1–4 (Table 1), based on the relative occurrence of WW and SS dinucleotides in 12 minor- and 12 major-GBS (Supplementary Table S1 and Figure S1). The Type 1 pattern represents the well-known WW/SS sequence pattern in which WW and SS preferentially occur in minor-GBS and major-GBS, respectively (Figure 1A). Type 2 nucleosomes follow a ‘mixed’ pattern, in which both WW and SS dinucleotides are more abundant in minor-GBS than major-GBS (Figure 1B). The Type 3 pattern is opposite to the Type 2 pattern in which both WW and SS are more abundant in major-GBS than minor-GBS (Figure 1C). The Type 4 pattern is inverse to Type 1 with WW and SS dinucleotides preferentially occurring in major- and minor-GBS respectively (Figure 1D). For the sake of simplicity, nucleosomes with the Type 1 pattern are denoted as WW/SS nucleosomes, whereas those with the Type 4 pattern are denoted as anti-WW/SS nucleosomes.

**Figure 2.**
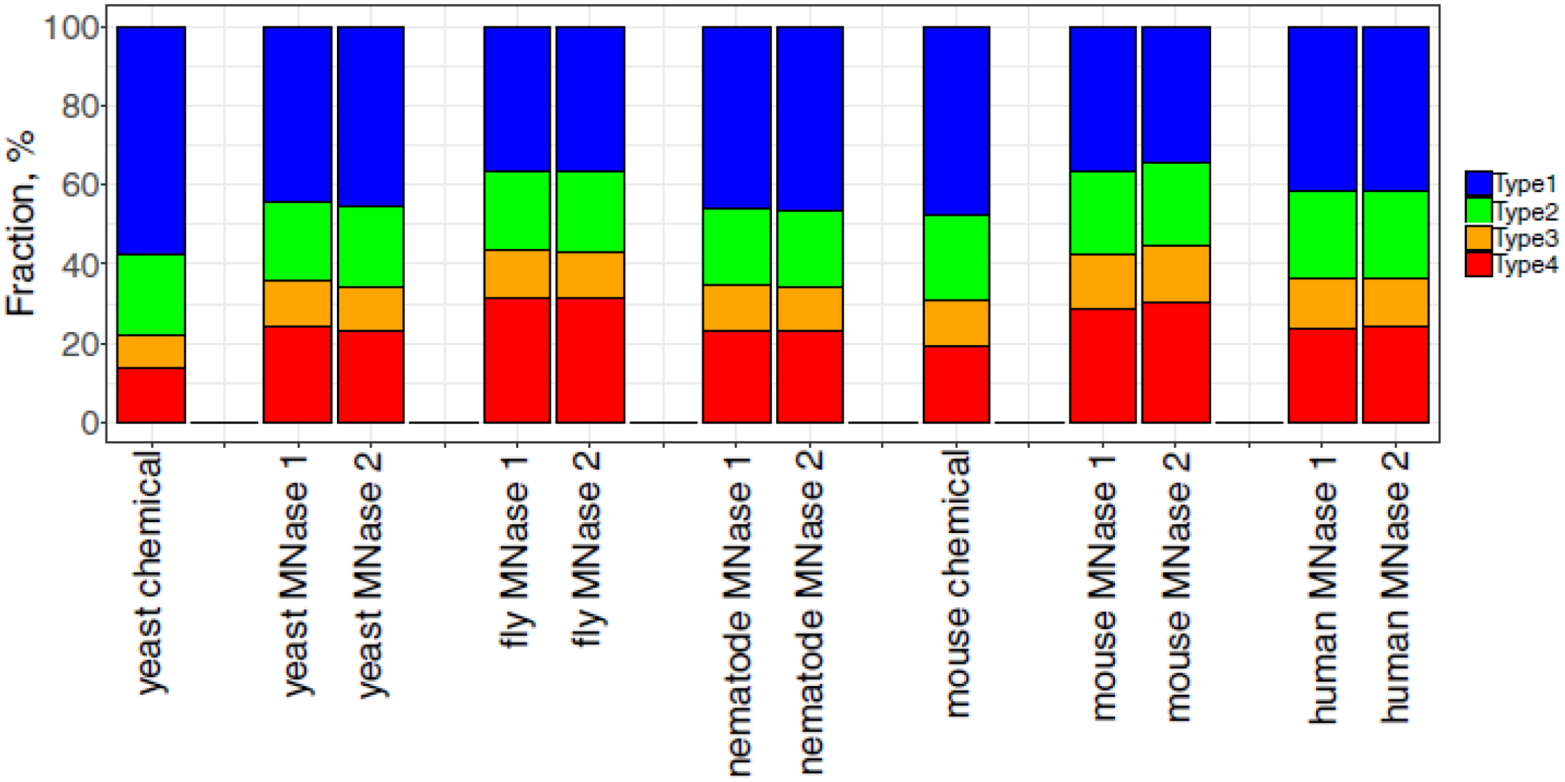
Comparison of the fractions of nucleosomal DNA patterns in eukaryotes. Shown is the fractions of four sequence patterns in nucleosomal DNA from yeast, nematode, fruit flies, mice and humans. For chemical mapping data, the ‘unique’ maps of nuleosomal dyad positions were taken from literature (10,13) and the corresponding 147-bp NCP fragments were used in this study. For the paired-end MNase mapping data, the two biological replicates or relevant datasets were taken from literature and 147-bp NCP fragments in these datasets were used for analysis. The fractions of sequence patterns were calculated for each dataset (Supplementary Table S4).

We have identified all four types of sequence patterns in datasets (Supplementary Tables S2 and S3) from yeast, fruit flies, nematodes, mice and humans (Figure 1 and Supplementary Figures S2–7). Our analysis has led to several interesting observations. First, since the datasets were produced by different methods (i.e. the MNase-Seq method or the chemical method), the presence of anti-WW/SS nucleosomes is independent of nucleosome mapping methods. Second, anti-WW/SS nucleosomes occur in all chromosomes (Supplementary Table S4), indicating that they are widespread in the genomes. Third, except for the yeast dataset obtained by chemical mapping, the fraction of WW/SS nucleosomes is less than 50% of all nucleosomes (Figure 2), indicating that the well-known WW/SS sequence pattern is not predominant. Fourth, the fractions of Type 1 and Type 4 nucleosomes vary substantially. For instance, the fractions of the four types of nucleosomes are 36–57% (Type 1), 20–22% (Type 2), 8–13% (Type 3) and 13–31% (Type 4), respectively (Supplementary Table S4). Since Type 2 and Type 3 nucleosomes have relatively small changes among datasets, we were prompted to develop a measure, ΔNPS ( = Type 1 (%) – Type 4 (%)), to gauge the difference between Type 1 and Type 4 nucleosomes (Supplementary Figure S8). A ΔNPS value represents the relative abundance of WW/SS *versus* anti-WW/SS nucleosomes in a given genomic region.

Taken together, anti-WW/SS nucleosomes accounting for 13–31% of total nucleosomes exist in all eukaryotes examined and are widespread across the genomes. We next sought to understand how anti-WW/SS nucleosomes are distributed in the genomes.

### Anti-WW/SS nucleosomes are enriched in mammalian genes and associated with transcription

**Figure 3.**
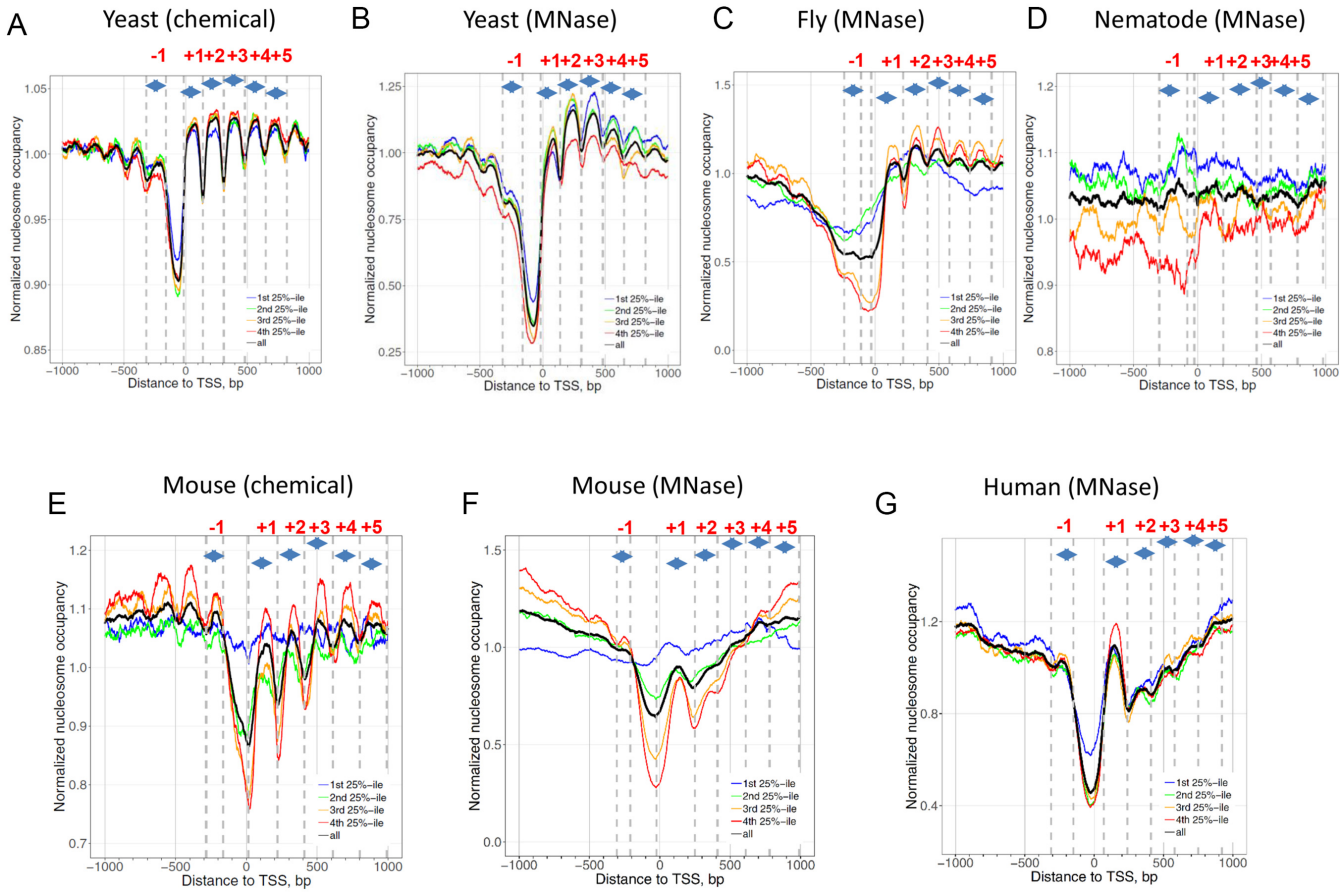
Nucleosome occupancy profiles around TSS of yeast (**A** and **B**), fly (B), nematode (**C**), mouse (**D** and **E**) and human (**F**) genes. There are two separate nucleosome datasets for yeast and mice, derived from chemical mapping (A and D) and MNase-Seq mapping (B and E), respectively. Nucleosome occupancy signals ±1 kb of verified TSSs are separated into quartiles based on transcriptional levels (Supplementary Table S5). The first 25%-ile represents the least active genes whereas the fourth 25%-ile represents the most active genes. The average nucleosome profile of all genes is shown in black. Nucleosomes −1 to +5 are demarcated by dashed lines and arrows, following the methods used in previous studies (77). These definable zones relative to the TSS (position 0) to which a nucleosome midpoint may be assigned are: −1, +1, +2, +3, +4 and +5 (see Supplementary Table S6 for nucleosome ranges). Note the nucleosome occupancy of the nematode dataset (D) looks noisier than other datasets probably due to low read coverage.

We started with genomic regions surrounding the transcriptional start sites (TSS) of genes that were separated into quartiles by transcriptional levels (Supplementary Table S5). As expected, nucleosome occupancy profiles in yeast reveal a well-established genomic pattern (48,49): the presence of an ∼200-bp nucleosome-depleted region (NDR), flanked by phased nucleosomes, which form a highly regular array extending into gene bodies (Figure 3A and B). The phased nucleosomes, named as nucleosome -1, +1, +2, +3, +4 and +5, are organized relative to TSS (50,51) (Supplementary Table S6).

Overall, the regularity of the nucleosome arrays depends on species, transcription levels and nucleosome mapping methods (Figure 3). First, nucleosome arrays in yeast are more regular than their counterparts in other species. Second, as transcriptional frequencies go higher, the nucleosome arrays tend to be more regular (compare red lines with blue lines). Third, nucleosomes mapped by the chemical method tend to more regularly positioned than those mapped by the MNase-Seq method (compare Figure 3A and B with Figure 3E and F), presumably because the hydroxyl-cleavage-based chemical method overcomes the sequence specificity of MNase (2,3) and is able to map nucleosomes at a higher resolution (6).

**Figure 4.**
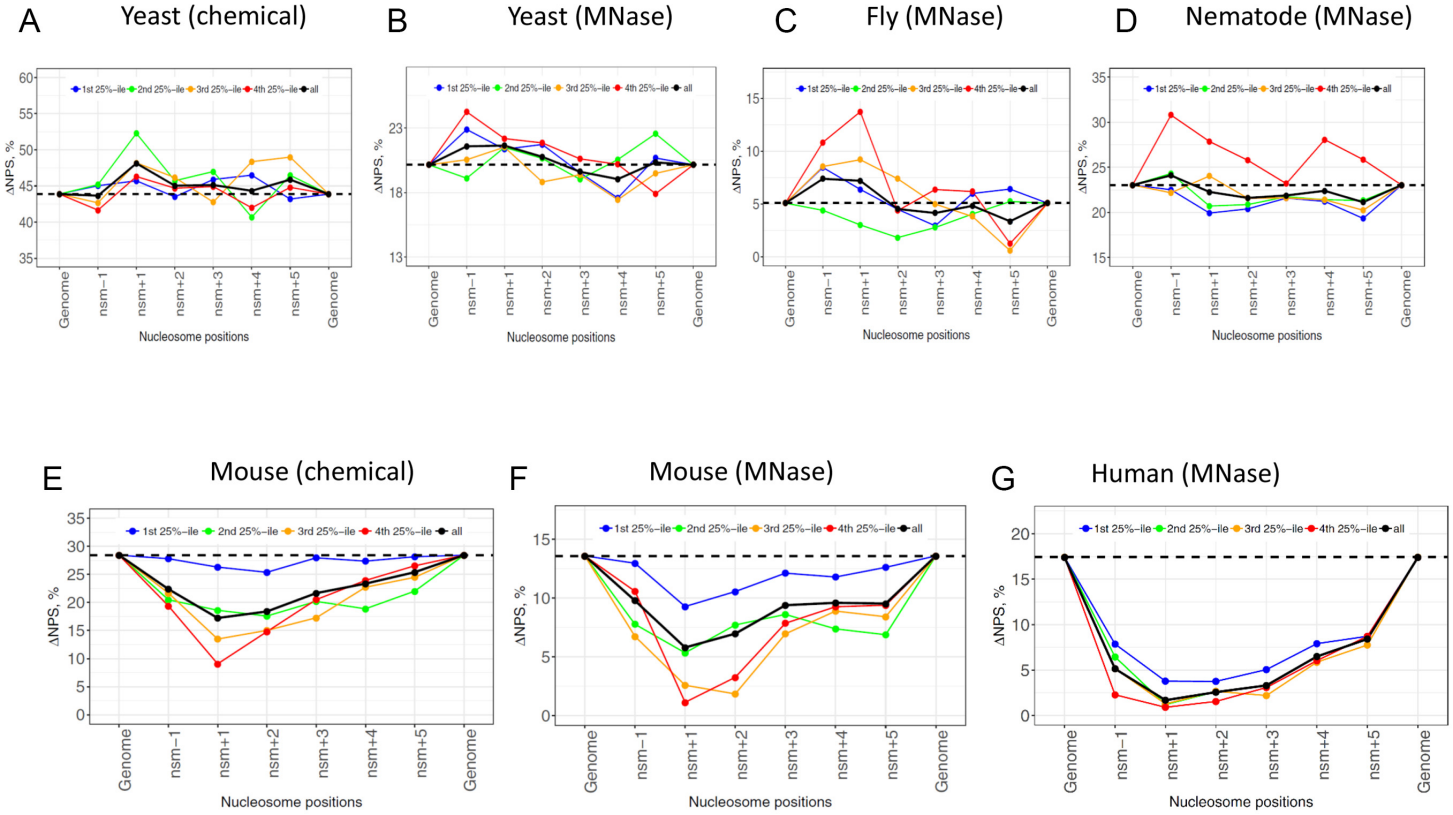
Nucleosome ΔNPS values for genes separated into quartiles by transcriptional frequencies. The first 25%-ile represents the least active genes whereas the fourth 25%-ile represents the most active genes. The ΔNPS values for all genes are shown in black. The genomic ΔNPS values are denoted by dashed lines.

Comparison of nucleosomal ΔNPS value for all genes across the species reveals some interesting patterns. First, in the non-mammalian genes, the ΔNPS values appear close to their genomic ΔNPS (see black lines and dashed lines in Figure 4A–D), suggesting that the relative abundance of Type 1 and Type 4 nucleosomes has little change. This pattern is confirmed by additional datasets in flies (Supplementary Figure S9A and B) and nematodes (Supplementary Figure S10A and B). Consistently, quantitative analysis reveals a small difference between nucleosomal ΔNPS (averaged over nucleosome −1 to +5) and genomic ΔNPS: <1% for yeast and 1–3% for flies and nematodes (Supplementary Table S7). In yeast, it has been shown that the fraction of Type 1 nucleosomes is significantly more than that of Type 4 nucleosomes with the difference ranging from ∼20% (MNase-seq set) to ∼45% (chemical set). This difference varies little between promoters and other regions in the yeast genome. Note that our results differ from a previous study by Ioshikhes and colleagues (29), in which a small set (<10 000) of single-end H2A.Z nucleosomal DNA sequences, compared to >4 million fragments in the present study (Supplementary Table S3), were used to analyze the positive or negative correlation with the WW/SS pattern. They found that the number of promotor nucleosomes following the anti-WW/SS pattern was higher or close to the number of nucleosomes following the well-known WW/SS pattern. Because the single-end sequencing method is unable to provide accurate estimations of nucleosome dyads, in our view, the observed discrepancies are likely due to the different methods, as well as different quality and size of datasets used in both studies.

Second, in mammalian genes, the ΔNPS values of nucleosome −1 to +5 tend to be lower than the genomic ΔNPS, with the lowest point at nucleosome +1 (black lines in Figure 4E-G), indicating that the relative abundance of Type 1 and Type 4 nucleosomes have changed. The averaged nucleosomal ΔNPS values exhibit a substantial difference, 5–11%, from the genomic ΔNPS value, which is much higher than that of non-mammals, 0–3% (Supplementary Table S7). Detailed analysis on the ΔNPS values of nucleosome +1 in the mouse chemical dataset shows that the fraction of Type 1 nucleosomes is decreased from 47 to 41%, whereas the fraction of Type 4 nucleosomes is increased from 19 to 24% (Supplementary Table S8). A similar trend is seen in the mouse and human MNase datasets (Supplementary Table S8), clearly illustrating that the fraction of Type 1 nucleosomes is decreased whereas the fraction of Type 4 nucleosome is increased in nucleosome +1. This mammalian pattern (Figure 4E–G) is in marked contrast to the non-mammalian one (Figure 4A–D), and an analysis of statistical errors of both patterns shows that anti-WW/SS nucleosomes are indeed enriched in mammalian genes (Supplementary Figure S11). Note that the observed difference is not due to the selected 147-bp NCP fragments because this difference still holds in a separate study that includes all NCP fragments (Supplementary Figure S12).

Third, the change in ΔNPS values is more correlated with transcriptional levels in mammalian genes compared to non-mammalian ones (Figure 4). For example, in yeast, no clear change is seen for the nucleosomal ΔNPS values in highly (red line) and lowly (blue line) transcribed genes (Figure 4A–D). By contrast, in mammals, lowly transcribed genes tend to have higher ΔNPS values whereas highly transcribed genes tend to have lower ΔNPS values (Figure 4E–G). Again, the nucleosome +1 was used for illustration (Supplementary Table S8): the fraction of Type 1 nucleosomes is decreased from lowly transcribed genes (Group 1) to highly transcribed genes (Group 4). By contrast, the fraction of Type 4 nucleosomes is increased from lowly transcribed genes to highly transcribed genes. These results have established a clear link between RNA Pol II transcription levels and the rotational settings of nucleosomes in mammals, that is, nucleosomes are more likely to follow the well-known WW/SS pattern in lowly transcribed genes and the anti-WW/SS pattern in highly transcribed genes.

Overall, we have seen a drastic difference in the distribution of anti-WW/SS nucleosomes in non-mammalian *versus* mammalian genes. That is, anti-WW/SS nucleosomes are relatively increased in genes of the mammals as compared to the non-mammalian species. Moreover, the local increase of anti-WW/SS nucleosomes seems to be associated with RNA Pol II transcription.

### Periodic DNA patterns are missing in mammalian genes

**Figure 5.**
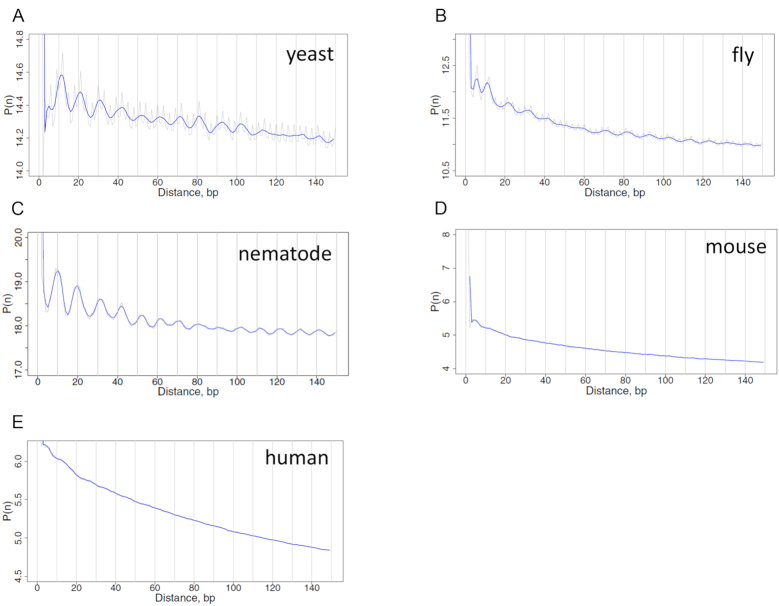
DAC function profiles for WW dinucleotides in yeast (**A**), fly (**B**), nematode (**C**), mouse (**D**) and human (**E**) DNA. Genomic fragments [−500 bp, +1000 bp] relative to verified TSSs (position 0) were used for analysis. Both raw (thin lines) and 3-bp running average (thick lines) values were plotted.

To check whether the observed local increase of anti-WW/SS is caused by underlying DNA patterns, we calculated DAC functions (46,47) of WW dinucleotides in the TSS-surrounding regions (i.e. between −500 bp and +1000 bp relative to TSS). Inspection of the DAC functions in non-mammals reveals a very regular structure, with peaks separated by ∼10 bp (Figure 5A–C). The same ∼10-bp periodicity is observed in the DAC functions of SS dinucleotides (Supplementary Figure S13A–C) and the DCC functions between WW and SS dinucleotides (Supplementary Figure S14A–C). Note that the DAC functions have the peaks at the distance of ∼10 × n bp (where *n* = 1, 2, …), whereas the DCC functions have the peaks at the distance of ∼10 × *n* + 5 bp (where *n* = 1, 2, …), indicating that WW (or SS) are in phase with each other, whereas WW and SS dinucleotides are out of phase. Our results suggest stable nucleosomes are likely to form with an optimal rotational setting in non-mammalian genes, which may explain small variations of the ΔNPS values observed for nucleosome −1 to +5 (see black lines in Figure 4A–D).

By contrast, both DAC and DCC functions in mouse and human genes exhibit no recognizable peaks (Figure 5D and E; Supplementary Figures S13D-E and S14D-E), indicating that the ∼10-bp periodic WW and SS patterns are missing in mammals, consistent with earlier studies (52). This lack of sequence periodicity in mammals is remarkable because it suggests that the local increase of anti-WW/SS is not due to intrinsic DNA sequence patterns but to *trans*-acting factors such as RNA Pol II. RNA transcription may facilitate the formation of anti-WW/SS nucleosomes that are intrinsically unstable, which in turn promotes gene transcription (see ‘Discussion’ section).

Taken together, we have observed a significant difference in DNA sequence patterns between mammalian and non-mammalian genes. The non-mammalian DNA exhibits an in-phase pattern of WW (or SS) dinucleotides and an out-of-phase pattern between WW and SS dinucleotides. By contrast, no periodic pattern is observed in mammals. Our data suggest that DNA sequence plays a distinctive role in the rotational settings of nucleosomes in non-mammals *versus* mammals around TSS.

### Anti-WW/SS nucleosomes are not over-represented in mammalian repeats

To examine if the enrichment of anti-WW/SS nucleosomes in mammals is context dependent, we focused on repetitive DNA elements. It is because that up to 40–50% of a mammalian genome contain repeat sequences derived from transposable elements (53), including elements with LTR, SINE and LINE. Many repetitive sequences occur in heterochromatic regions (54) that are characterized with high levels of condensation throughout the cell cycle (55), low rates of meiotic recombination (56) and the ability to silence gene expression (57). We therefore hypothesize that the fraction of anti-WW/SS nucleosomes in repeats would differ from that in genic regions (Supplementary Figure S8).

**Figure 6.**
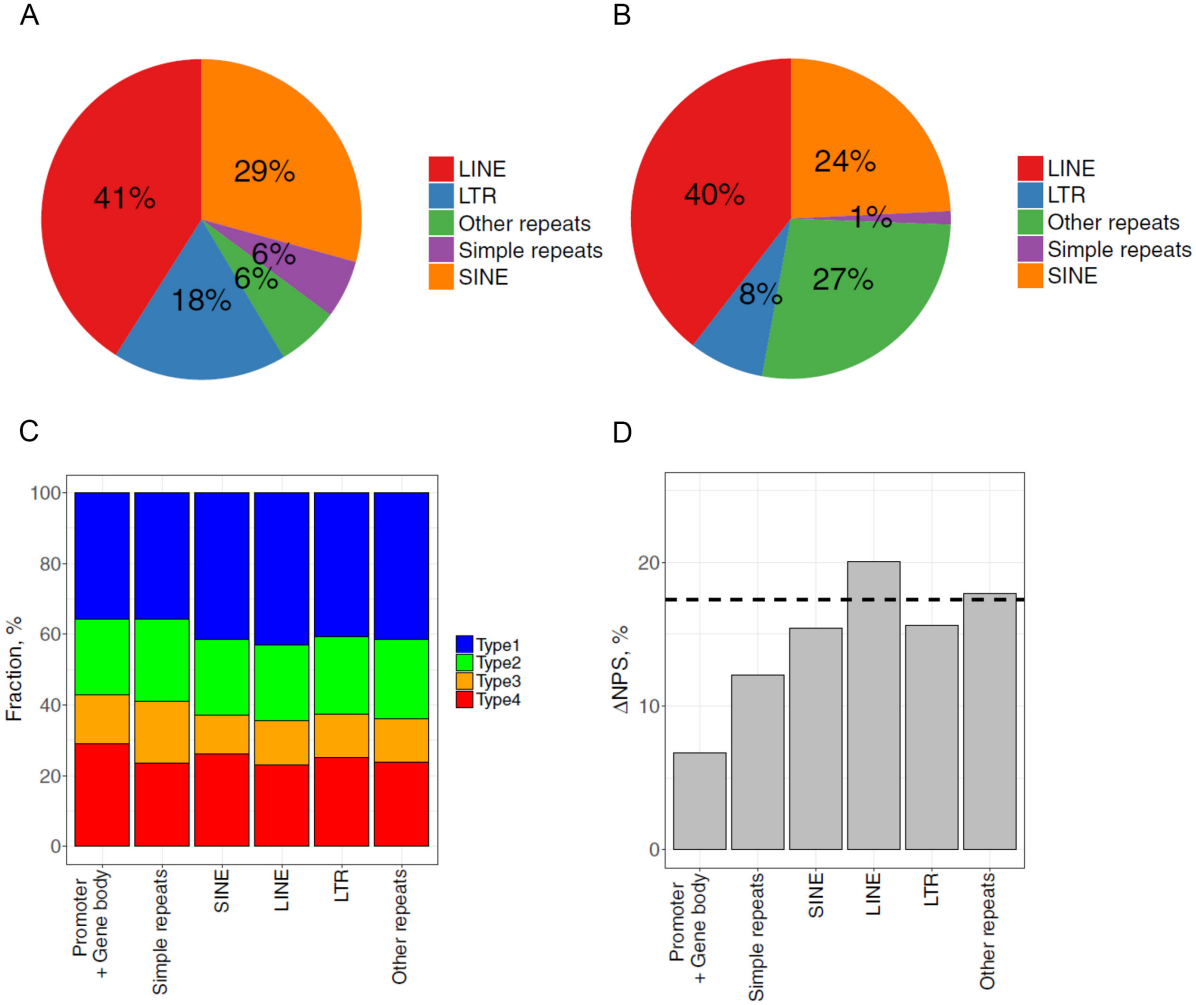
Fractions of DNA sequence patterns and ΔNPS values in human repeat families. (**A**) Fraction of repeat families in human genome. The fractions were taken from literature (78). (**B**) Fraction of 147-bp human nucleosomes residing in each repeat family. (**C**) Fraction of four types of nucleosomes in each repeat family. (**D**) Nucleosome ΔNPS values in genic and repetitive DNA regions. The genomic ΔNPS value is indicated by dashed lines.

We first checked the fraction of repeat families in the human genome (Figure 6A; Supplementary Tables S9 and S10) and compared with the fractions of nucleosomes residing in these families (Figure 6B). Although 18 and 6% of human repeats are LTR and simple repeat elements, only 8 and 1% of NCP fragments are found in these elements, respectively, indicating that nucleosomes are depleted in these families. By contrast, no apparent depletion of nucleosomes is observed in LINE and SINE elements (41 versus 40% for LINE and 29 versus 24% for SINE) (Figure 6A and B).

Then we calculated the fractions of four sequence types and the ΔNPS value across repeat families (Figure 6C and D) and found that ΔNPS values in all families are higher than that in genic region. In particular, SINE- and LINE-bearing nucleosomes have ΔNPS values close to the genomic value (Figure 6D). Similar observations are seen in mouse repeats (Supplementary Table S11 and Supplementary Figure S15), indicating that anti-WW/SS nucleosomes are not over-represented in mammalian repeats. Examination of the DAC functions of WW dinucleotides in human LINE and SINE elements reveals an ∼10-bp periodicity (Supplementary Figure S16), which is absent in genic regions (Figure 5D and E), suggesting that repetitive DNA is organized differently from DNA in genic regions.

### Chromatin remodelers influence the fraction of anti-WW/SS nucleosomes

**Figure 7.**
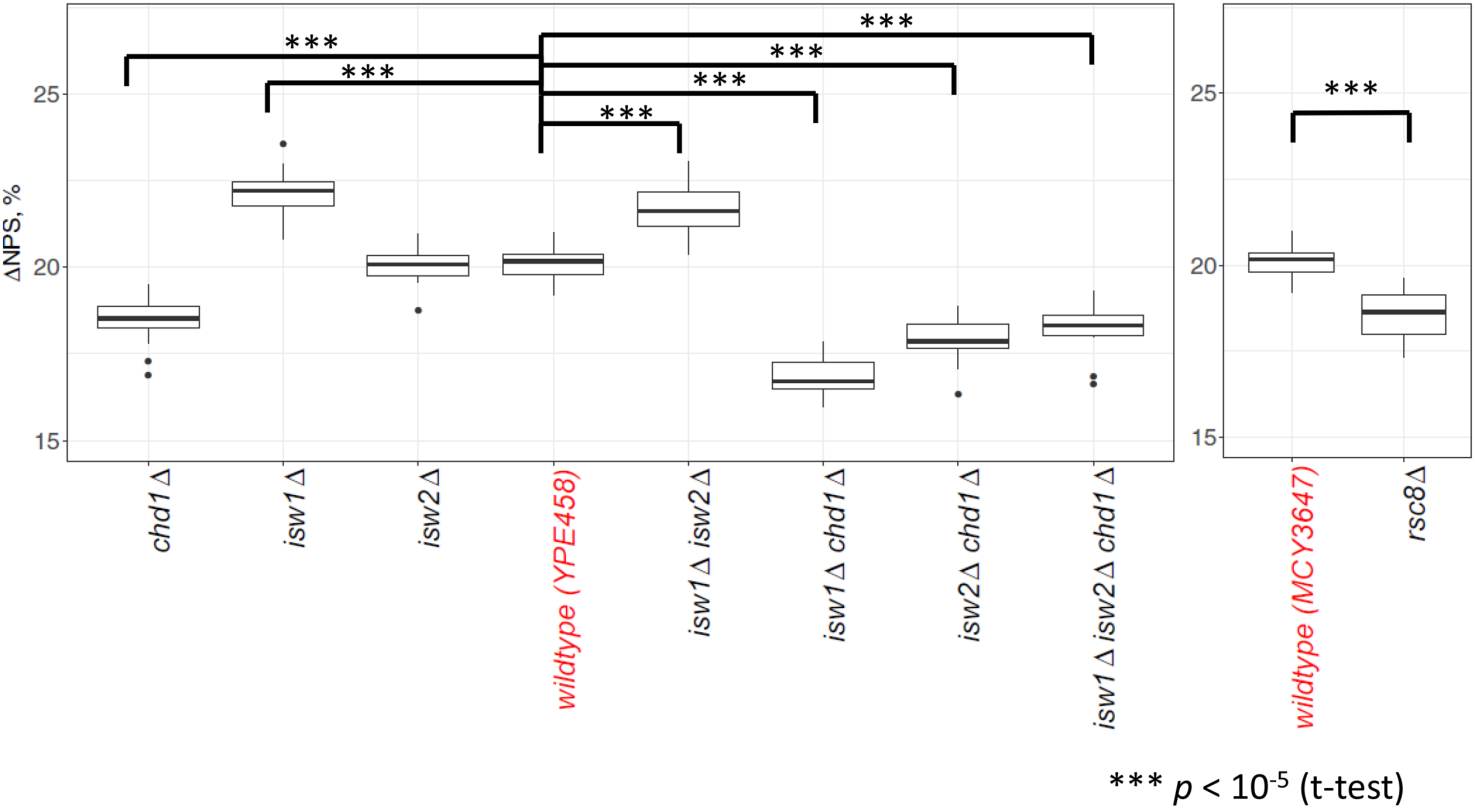
Genomic ΔNPS values in yeast WT and mutant strains. ΔNPS values were calculated by chromosomes and shown in a box-whisker plot, with the mean representing the genomic ΔNPS. The genomic ΔNPS values of WT strains are used as reference points to compare with those of mutant strains (Supplementary Table S12) in which one or more chromatin remodeler genes are knocked out (50).

At last, we investigated if other *trans*-acting factors such as chromatin remodeling complexes can influence the abundance of anti-WW/SS nucleosomes. To this end, yeast mutant strains in which single or multiple chromatin remodeling complexes have been deleted were used for analysis. These mutant strains include four single mutants (*isw1Δ, isw2Δ, chd1Δ* and *rsc8Δ*), three double mutants (*isw1Δ isw2Δ, isw1Δ chd1Δ* and *isw2Δ chd1Δ*), and one triple mutant (*isw1Δ isw2Δ chd1Δ*) (37,38). From the fraction of four sequence types (Supplementary Figure S17) and genomic ΔNPS value (Supplementary Table S12) of each mutant, it is clear that all mutants except *isw2Δ* have significantly different ΔNPS values from the WT strain (Student's *t*-test *P* < 10^−5^) (Figure 7), with the average deviation being 1.85% (Supplementary Table S12).

**Figure 8.**
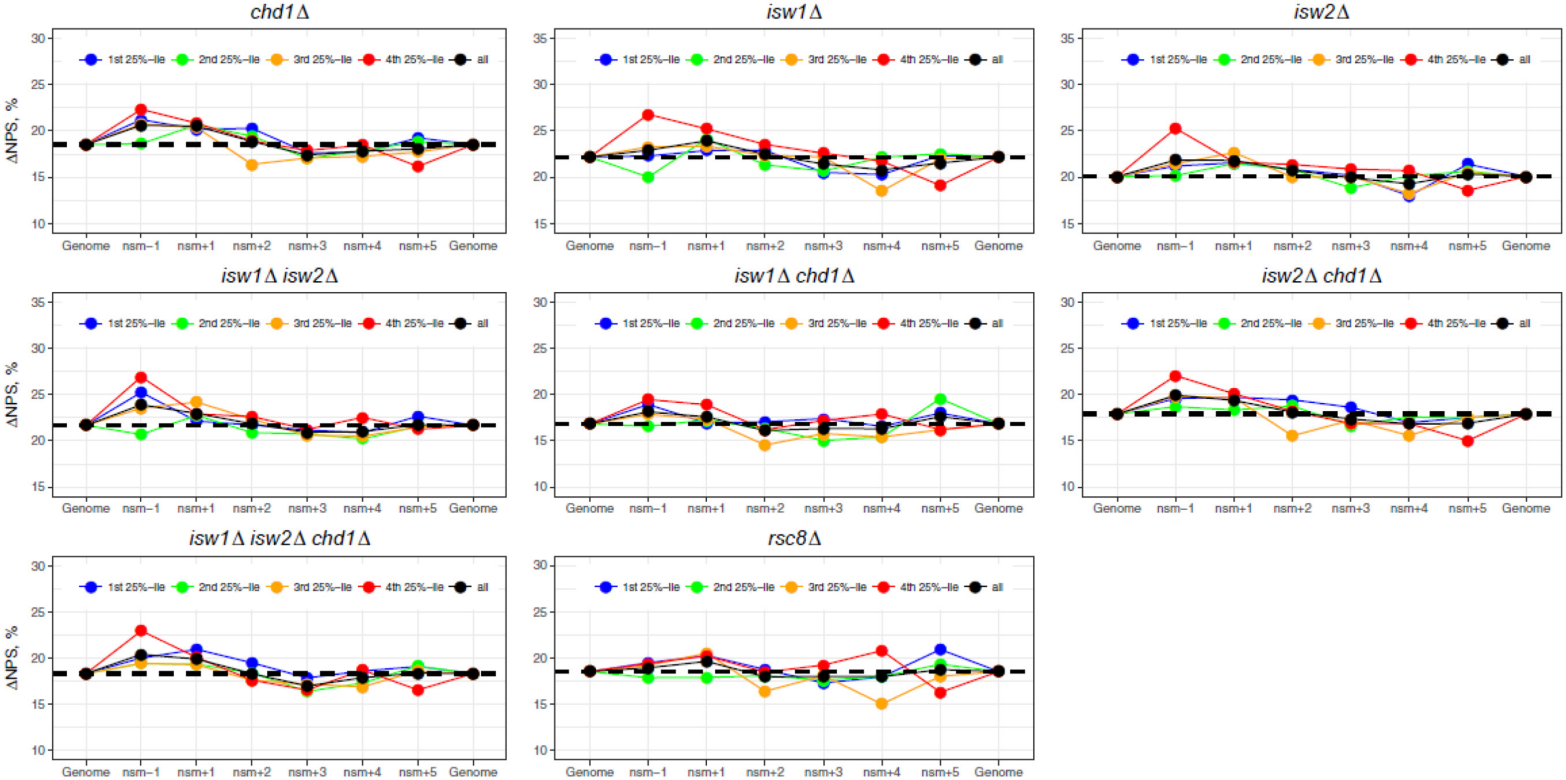
ΔNPS values of promoter and downstream nucleosomes in yeast mutant strains. For the sake of comparison, the ranges of nucleosomes are the same for both the WT and mutant strains (Supplementary Table S6). Other notations follow Figure 3.

The double mutant strain *isw1Δ chd1Δ* was used for detailed analysis because nucleosome phasing is disrupted (Supplementary Figure S18A), as shown before (37). The genomic ΔNPS value of this strain is 16.84%, which is 3.3% smaller than that of the WT strain, 20.14% (Supplementary Table S12), suggesting that the deletion of both ISW1 and CHD1 leads to an increase of anti-WW/SS nucleosomes. However, the nucleosome ΔNPS profile around TSS is very similar to that of WT strain (Supplementary Figure S18B and Figure 4A), indicating that anti-WW/SS nucleosomes are not enriched in the genic regions, although the overall fraction of anti-WW/SS nucleosomes is increased. Similar patterns are seen in other mutants, e.g., *chd1Δ isw1Δ chd1Δ, isw2Δ chd1Δ* and *isw1Δ isw2Δ chd1Δ* (Figure 8). Our data suggest that chromatin remodeler(s) have an impact on the fraction but not on the distribution of anti-WW/SS nucleosomes in non-mammals. However, it remains to be determined if this observation holds for mammals.

## DISCUSSIONS

### A model for *cis* and *trans* determinants of rotational positioning of nucleosomes

**Figure 9.**
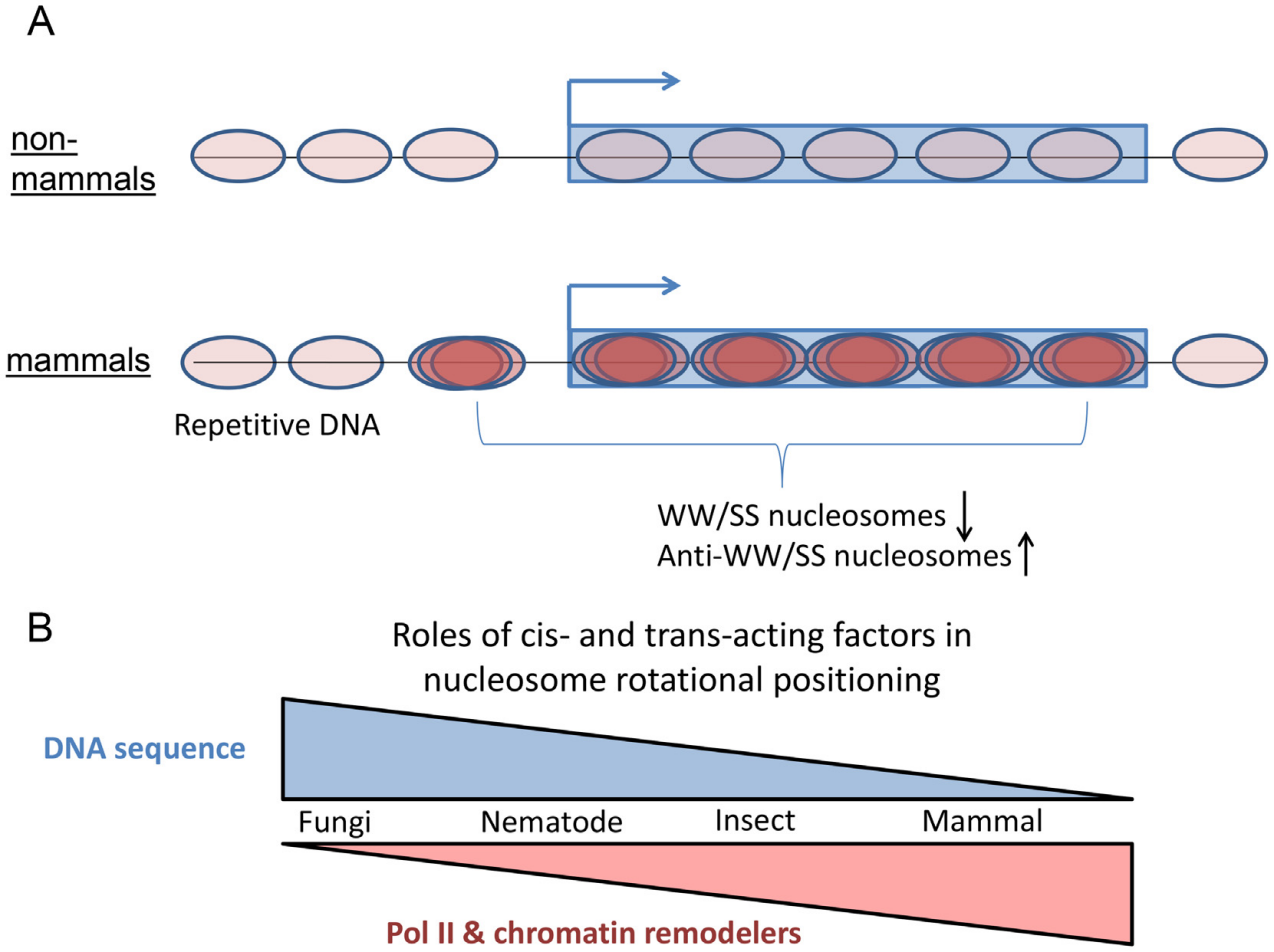
Models for the roles of *cis* and *trans* factors in rotational positioning of nucleosomes. (**A**) Species-specific distribution of anti-WW/SS nucleosomes. In mammals, the fraction of anti-WW/SS nucleosomes in nucleosomes −1 to +5 is increased and the fraction of WW/SS nucleosomes is decreased, suggesting that these nucleosomes are unstable (represented by fuzziness of nucleosome positioning). This change is not seen in non-mammalian genes. Nucleosomes −1 to +5 in mammals are depicted in dark brown and other nucleosomes (including those residing in mammalian repetitive DNA) are depicted in light brown. (**B**) Distinct role of *cis* and *trans* factors in rotational positioning of nucleosomes. Both cis-acting factors (e.g. DNA sequences) and *trans*-acting factors (e.g. chromatin remodeling complexes and RNA Pol II) affect rotational settings of nucleosomes. In non-mammals, DNA sequence plays a more important role than RNA Pol II because (i) the ∼10-bp periodic WW (or SS) patterns are pronounced; (ii) no clear change in the ΔNPS values is seen between highly and lowly transcribed genes. By contrast, in mammalian genes, RNA Pol II plays a more important role because (i) the ∼10-bp periodicity is diminished, and (ii) a clear change in ΔNPS values is seen between highly and lowly transcribed genes. Chromatin remodelers also have an impact on the rotational setting of nucleosomes in non-mammals, but this effect is unclear in mammals.

We have conducted a comprehensive analysis of WW/SS-based sequence patterns for nucleosomes in five eukaryotes. Our work demonstrates that nucleosomes with the anti-WW/SS pattern are widespread in eukaryotic genomes. Interestingly, these nucleosomes are distributed differently in non-mammals (e.g. yeast, nematodes and fruit flies) *versus* in mammals (e.g. mice and humans). Specifically, anti-WW/SS nucleosomes are enriched in the promoter and genic regions of mammalian genomes but not of non-mammalian genomes (Figure 9A). We have further analyzed the impact of various *cis*- and *trans*-acting factors on the fraction and the distribution of anti-WW/SS nucleosomes. In light of these findings, we propose a model for the distinct role of *cis* and *trans* factors in the rotational positioning of nucleosomes between non-mammals and mammals (Figure 9B).

Based on our model, the rotational positioning of nucleosomes can be influenced by *cis*-acting factors (DNA sequence patterns) and trans-acting factors (chromatin remodeling complexes and RNA Pol II) in both non-mammals and mammals. However, the relative importance of these factors differ. In non-mammals, the *cis*-acting factors play a more important role than *trans*-acting factors because (i) genomic DNA exhibits sequence patterns with an ∼10-bp periodicity (Figure 5A–C; Supplementary Figures S13A–C and S14A–C), and (ii) the fraction of anti-WW/SS nucleosomes varies little across the genomes (Figures 4A–D and 8). By contrast, the *trans*-acting factors such as RNA Pol II appears to be more important in mammals, due to a lack of periodic sequence patterns (Figure 5D and E; Supplementary Figures S13D-E and S14D-E), and the local increase of anti-WW/SS nucleosomes in mammalian promoters and genic regions (Figure 4E–G).

It is worth noting that overall, periodic WW (or SS) patterns appear to be very strong in yeast, become weaker in nematodes and flies and completely disappear in mice and humans (Figure 5 and Supplementary Figures S13 and S14). This implies that the importance of *cis*-acting factors in the rotational positioning of nucleosomes decreases in evolution, especially in promoter and genic regions. However, the periodic sequence patterns are preserved in mammalian repetitive DNA elements (Supplementary Figure S16) and anti-WW/SS nucleosomes are not over-represented (Figure 6D). These data suggest that molecular mechanisms governing the rotational settings of nucleosomes in mammals are context dependent.

Recenlty, Tompitak *et al.* have found a large difference in the intrinsically encoded nucleosome affinity at promoter regions of unicellular *versus* multicellular eukaryotes (58). Unlike the NDR-encoded in unicellular eukaryotes (e.g. yeast), promoters in multicellular eukaryotes (e.g. humans) encode nucleosome-attracting regions (NAR), which is a universal feature of multicellular life. Two interesting hypotheses have been proposed to account for the presence of NAR in humans: one is to suppress gene transcription and facilitate cell differentiation, whereas the other is to retain nucleosomes in the germline to pass on epigenetic information. Together with our findings, we hypothesize that two different codes for nucleosome positioning evolve independently: the code for translational positioning (represented by intrinsic nucleosome affinity) is weak in yeast and becomes increasingly strong late in evolution, whereas the code for rotational positioning (represented by WW/SS patterns) is strong in yeast and gradually disappears late in evolution.

### Implications of anti-WW/SS nucleosomes for RNA Pol II transcription

The interactions between RNA Pol II transcription and nucleosomes have been extensively studied since the discovery of nucleosomal barrier (59,60). Early work has established that the nucleosome presents a strong barrier to transcription *in vitro*, and the nature of this barrier has been studies by structural, biochemical and biophysical studies (61,62). However, the questions of how the Pol II machinery efficiently overcomes nucleosomes and transcribes through gene body *in vivo* remain unclear. Various *trans* factors modulating the nucleosomal barrier have been proposed (60), including histone modifications, histone variant replacement, histone chaperones and nucleosome remodelers. Our work suggests that *cis* factors such as DNA sequence also contribute to modulation of nucleosomal barrier, especially in mammals (see below).

As shown above, anti-WW/SS nucleosomes are enriched in promoters and genic regions of mammals, and this enrichment is increased with the transcriptional levels. One possible explanation is that because of the intrinsic instability of nucleosomes with respect to rotational settings, RNA Pol II transcription is promoted. However, the formation of anti-WW/SS nucleosomes is not energetically favorable because GC-containing dinucleotides in the minor-GBS do not have favorable interactions with the ‘sprocket’ arginine residues in histones (see ‘Introduction’ section). In addition, the enrichment of this type of nucleosomes is not seen in highly transcribed genes in non-mammals (Figure 4A–D). An alternative explanation is that RNA Pol II transcription helps to position anti-WW/SS nucleosomes that are thermodynamically unstable. In these nucleosomes, the presence of SS dinucleotides in the minor-GBS weakens DNA-histone interactions, which may facilitate the unwrapping of DNA from nucleosomes (63), thereby promoting RNA Pol II transcription. This tendency may also help explain histone eviction observed in highly transcribed genes (64,65).

### Rotational positioning and binding of transcriptional factors in mammalian repeats

Repetitive DNA regions in human are characterized with different chromatin organizations. For example, LTR/ERV elements are highly enriched in open chromatin regions (66), whereas Alu elements that belong to the SINE superfamily harbor two nucleosomes (67–69). Our analysis of the human nucleosome data, which were derived from lymphoblastoid cell lines (42), is in general agreement with these observations (Figure 6A and B).

A group of TFs known as pioneer factors (e.g., p53, Oct4, Sox2, Klf4 etc.) is able to interact with nucleosomal DNA (70–72) and many of them preferentially bind to repetitive DNA elements (73). We and other groups have shown that the rotational positioning of nucleosomes is critical for TF binding including p53 (70,74). We have found that an Alu-residing nucleosome, if taking the optimal rotational setting, help to expose the putative binding sites of p53 (75). The present study has demonstrated that nucleosomes are likely to take the optimal rotational setting in human repeats including SINE/Alu elements (Figure 6D), suggesting that many p53 binding sites in Alu elements are likely to be exposed. This is also in agreement with our recent findings that a large amount (∼40%) of p53 binding sites occurs in various repeats including Alu (76). Further analyses are needed to address the biological roles of anti-WW/SS nucleosomes in repeats to see if, in these cases, TF binding sites are buried inside of nucleosomes and inhibitory for TF binding, as we suggested for p53 (74). Understanding the rotational positioning of nucleosomes in human repeats will shed new light on mechanisms regulating retrotransposon activities in a normal or diseased cellular context.

## Supplementary Material

gkz544_Supplemental_Files

## Notes

Present address: Gregory M. Wright, Department of Pharmacology, School of Medicine, University of Colorado (Anschutz Medical Campus), Aurora, CO 80045, USA.
