# Supplementary material for: The nucleosome position-encoding WW/SS sequence pattern is depleted in mammalian genes relative to other eukaryotes
Source: Nucleic Acids Res. 2019 Jun 19;47(15):7942–54. doi: 10.1093/nar/gkz544 (PMC6735720; doi:10.1093/nar/gkz544)
Supplement: gkz544_Supplemental_Files [file gkz544_supplemental_files.zip › CUI_Anti_NPS_Supplemental_materials_May_2019.pdf]

Supplementary Materials

The nucleosome position-encoding WW/SS sequence pattern is depleted in mammalian genes relative to other eukaryotes

Gregory M. Wright and Feng Cui*

Thomas H. Gosnell School of Life Sciences, Rochester Institute of Technology, 85 Lomb Memorial Drive, Rochester, NY 14623, USA

*To whom correspondence should be addressed:

Tel: +1 585 475 4115; Fax: +1 585 475 2398; Email: fxcsbi@rit.edu

Contents

Supplementary Figures 2

Figure S1 2
Figure S2 3
Figure S3 4
Figure S4 5
Figure S5 6
Figure S6 7
Figure S7 8
Figure S8 9
Figure S9 10
Figure S10 11
Figure S11 12
Figure S12 13
Figure S13 14
Figure S14 15
Figure S15 16
Figure S16 17
Figure S17 18
Figure S18 19

Supplementary References 20

A

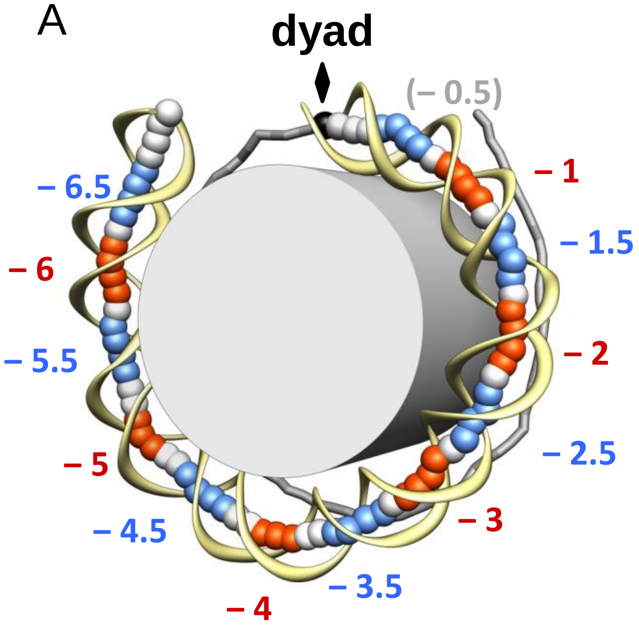

B

minor-groove bending sites

DNA position	SHL value
[5-8]	(SHL -6.5)
[15-18]	(SHL -5.5)
[26-29]	(SHL -4.5)
[37-40]	(SHL -3.5)
[47-50]	(SHL -2.5)
[57-60]	(SHL -1.5)

Major-groove bending sites

DNA position	SHL value
[10-14]	(SHL -6)
[21-23]	(SHL -5)
[32-34]	(SHL -4)
[42-45]	(SHL -3)
[52-55]	(SHL -2)
[62-65]	(SHL -1)

Figure S1. Locations of the minor- and major-groove bending sites (GBS) in nucleosomal DNA. (A) The crystal structure of the 1KX5 nucleosome core particle (NCP) with 147-bp long DNA (Davey et al. 2002) shown schematically: the DNA fragment is divided into two halves, separated by the dyad (black ball and arrow). The base-pair centers in the 'ventral' half are represented by large balls, and the sugar-phosphate backbone is shown by a yellow ribbon. For the 'dorsal' half of the nucleosome, the base-pair centers are connected by sticks. Minor- and major-groove bending sites are shown in blue and red, respectively. These sites are named by their superhelical locations (SHL). (B) The exact locations of minor- and major-GBS in the 'ventral' half of the nucleosomal DNA fragment are shown. The sites on the 'dorsal' half are symmetrical to their counterparts on the 'ventral' half with respect to the dyad (Cui and Zhurkin 2010, Supplemental Table S1). The minor-groove bending sites at SHL ±0.5 (in grey) are not included for analysis because DNA patterns are out of phase at these locations (Satchwell et al. 1986).

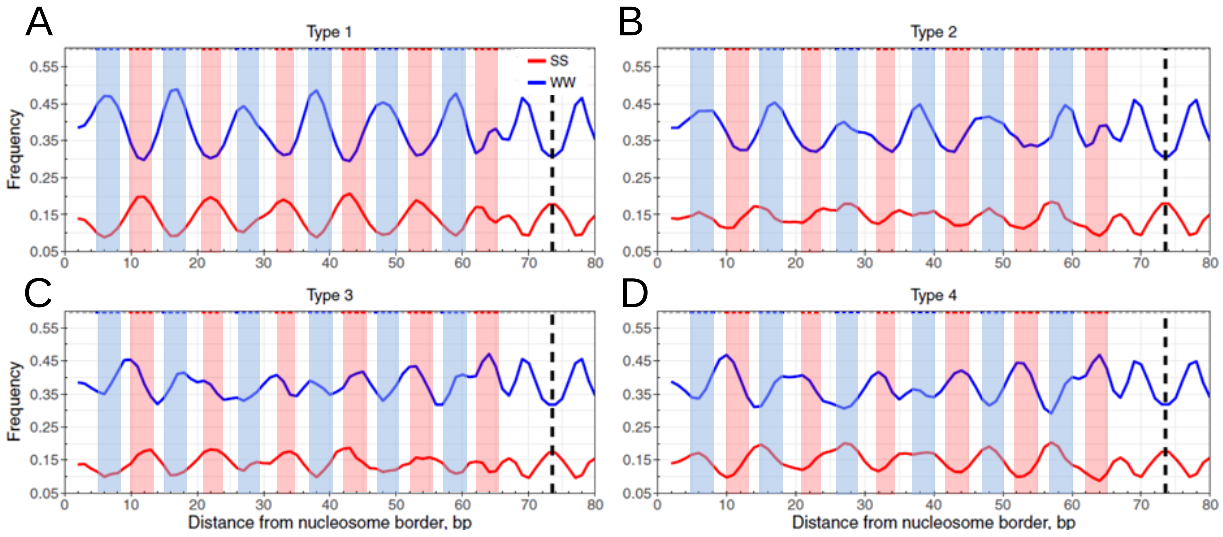

Figure S2. Four sequence patterns of yeast nucleosomal DNA mapped by a chemical method. The dyad positions of nucleosomes were published previously (Brogaard et al. 2010). Notations are the same as in Figure 1.

.

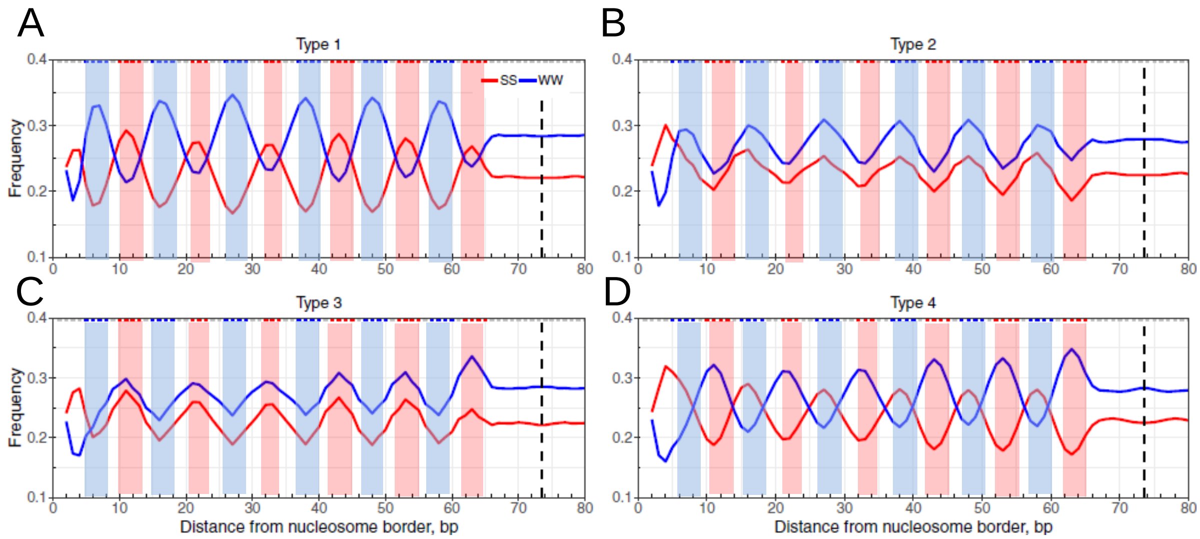

Figure S3. Four sequence patterns of fruit fly nucleosomal DNA mapped by MNase-Seq. The nucleosomal DNA fragments were sequenced by paired-end sequencing and the data were published previously (Chereji et al. 2015). Notations are the same as in Figure 1.

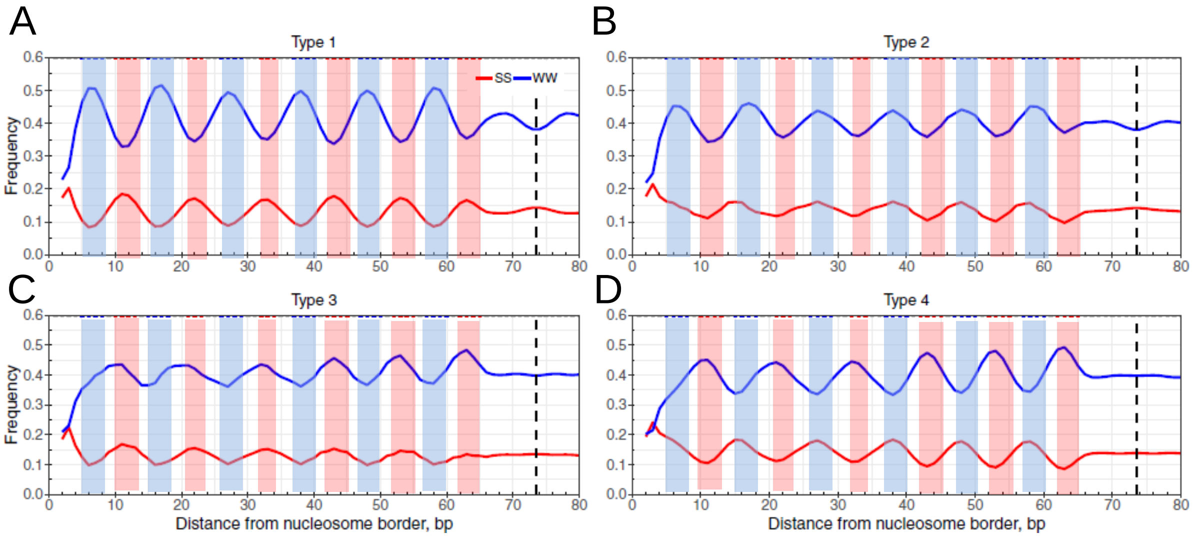

Figure S4. Four sequence patterns of nematode embryo nucleosomal DNA mapped by MNase-Seq. The nucleosomal DNA fragments were sequenced by paired-end sequencing and the data were published previously (Tabuchi et al. 2015). Notations are the same as in Figure 1.

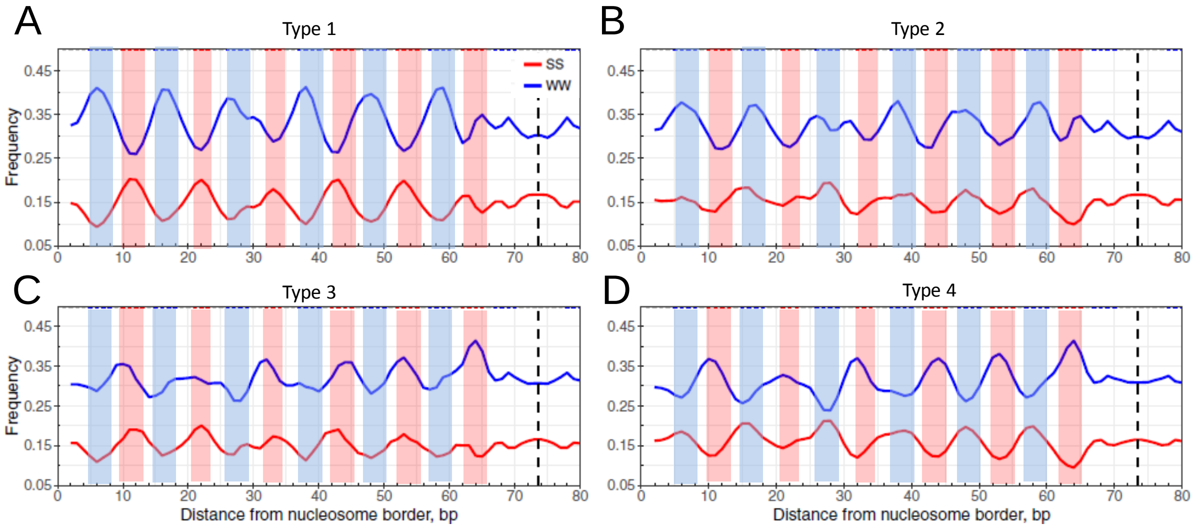

Figure S5. Four sequence patterns of mouse mESC nucleosomal DNA mapped by a chemical method. The dyad positions of nucleosomes were published previously (Voong et al. 2016). Notations are the same as in Figure 1.

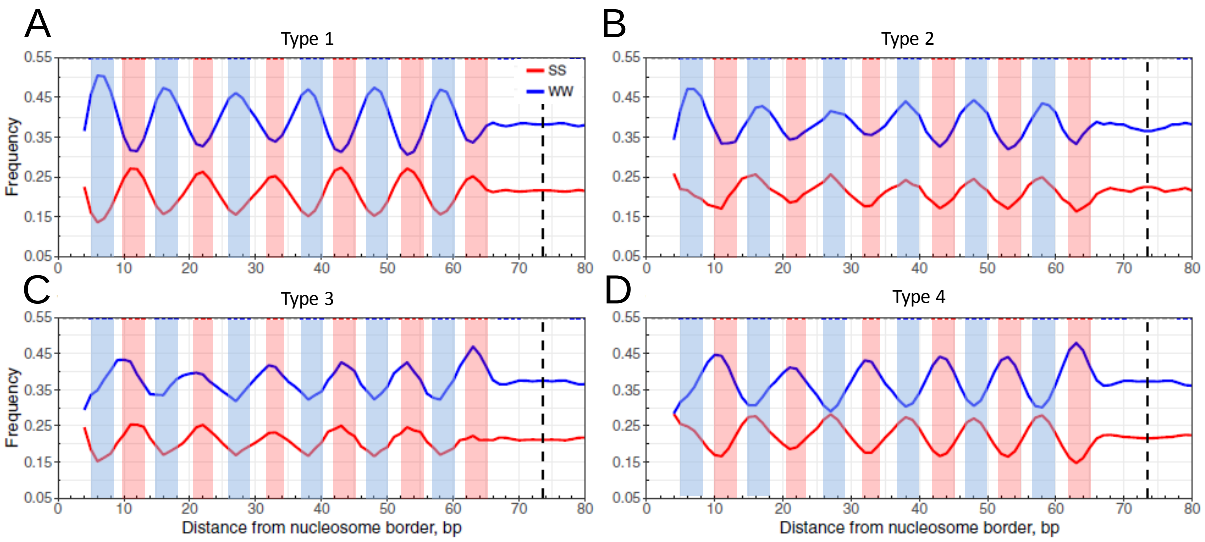

Figure S6. Four sequence patterns of mouse mESC nucleosomal DNA mapped by MNase-Seq. The nucleosomal DNA fragments were sequenced by paired-end sequencing and the data were published previously (Voong et al. 2016). Notations are the same as in Figure 1.

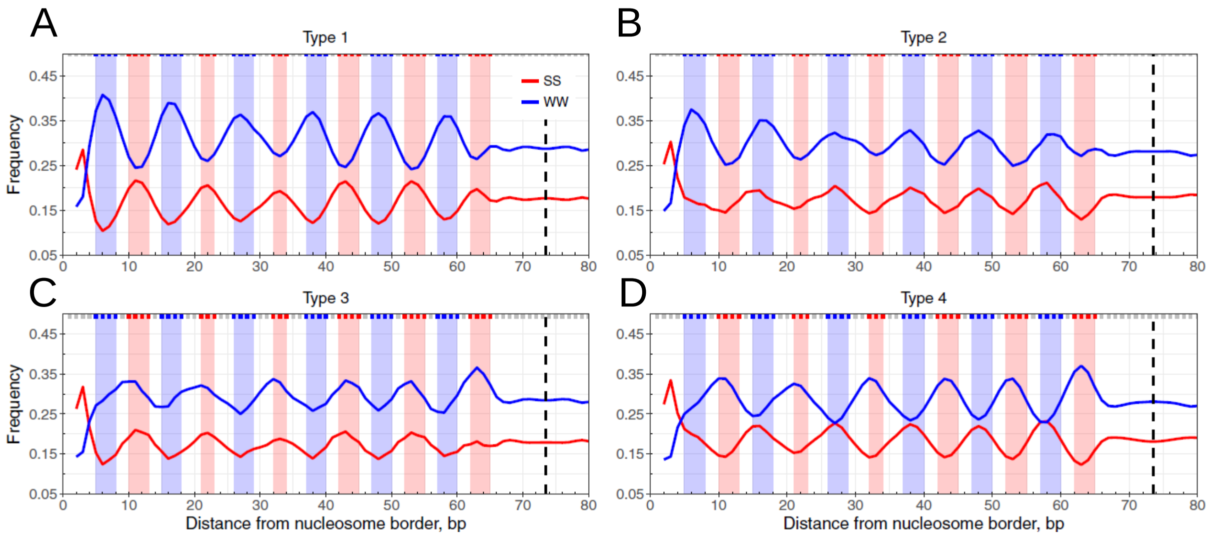

Figure S7. Four sequence patterns of human lymphoblastoid nucleosomal DNA mapped by MNase-Seq. The nucleosomal DNA fragments were sequenced by paired-end sequencing and the data were published previously (Gaffney et al. 2012). Notations are the same as in Figure 1.

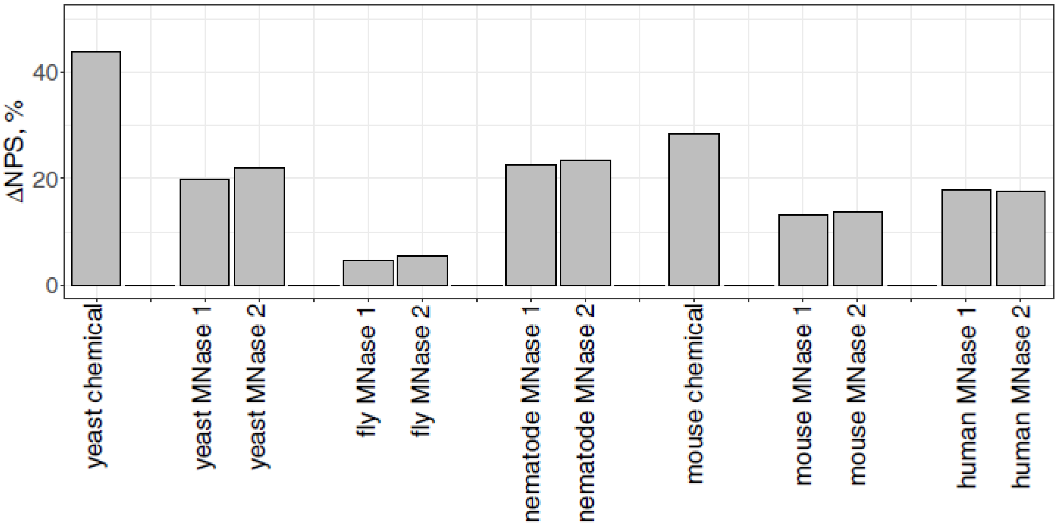

Figure S8. ΔNPS values of nucleosomal DNA datasets. The ΔNPS is calculated as the difference between Type 1 nucleosomes (%) and Type 4 nucleosomes (%). The details of fragment selection in each dataset are provided in Figure 2.

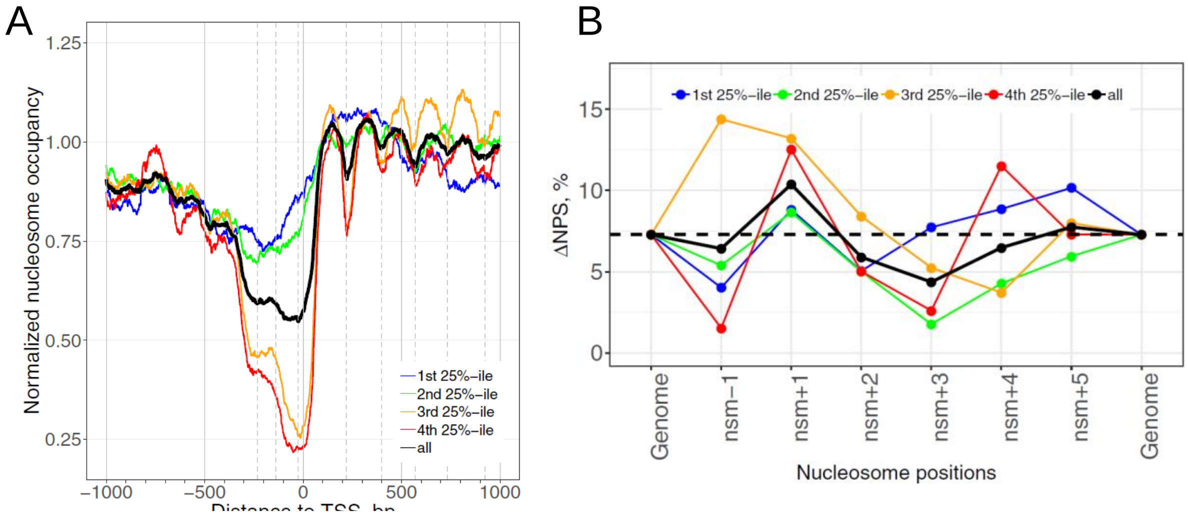

Figure S9. Nucleosome occupancy and ΔNPS values of fruit fly S2 cells. (A) Nucleosome occupancy profiles around TSS of fly. Nucleosomes mapped by MNase-Seq were taken from literature (Fuda and Lis 2015). Nucleosome occupancy signals ±1kb of verified TSSs are separated into quartiles by transcriptional frequencies based on RNA-seq data (Table S5). Notations are the same as Figure 2. (B) Nucleosome ΔNPS values in genes separated into quartiles by transcriptional frequencies. The ΔNPS values of all genes are shown in black. The genomic ΔNPS values are denoted by dashed lines.

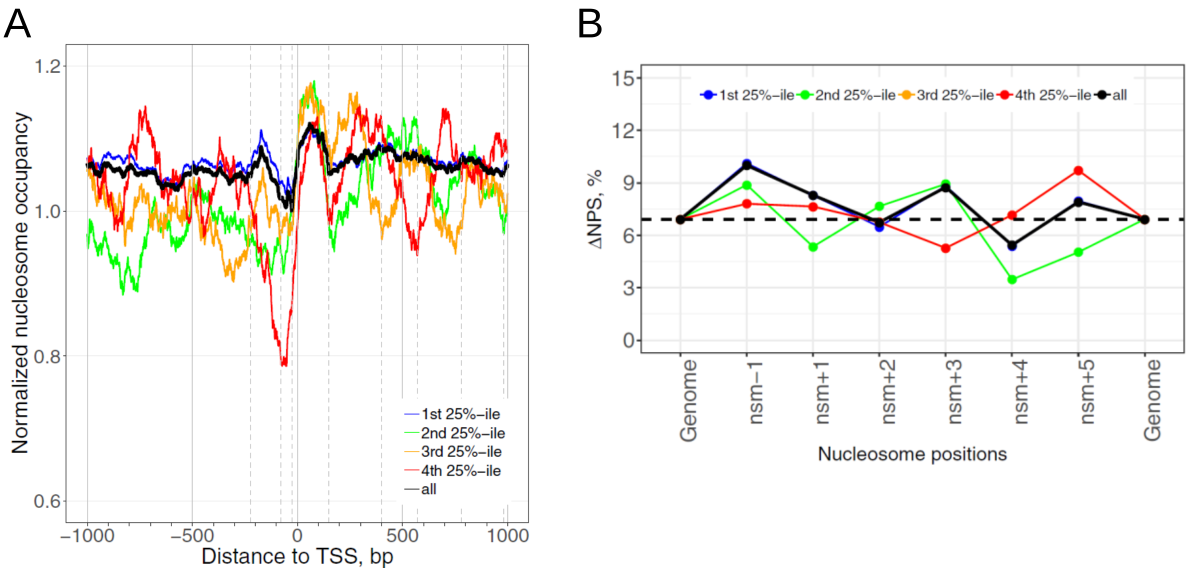

Figure S10. Nucleosome occupancy and ΔNPS values of nematode sperms. (A) Nucleosome occupancy profiles around TSS of fly. Nucleosomes mapped by MNase-Seq were taken from literature (Tabuchi et al. 2018). Nucleosome occupancy signals ±1kb of verified TSSs are separated into quartiles by transcriptional frequencies based on RNA-seq data (Table S5). Notations are the same as Figure 2. (B) Nucleosome ΔNPS values in genes separated into quartiles by transcriptional frequencies. The ΔNPS values of all genes are shown in black. The genomic ΔNPS values are denoted by dashed lines.

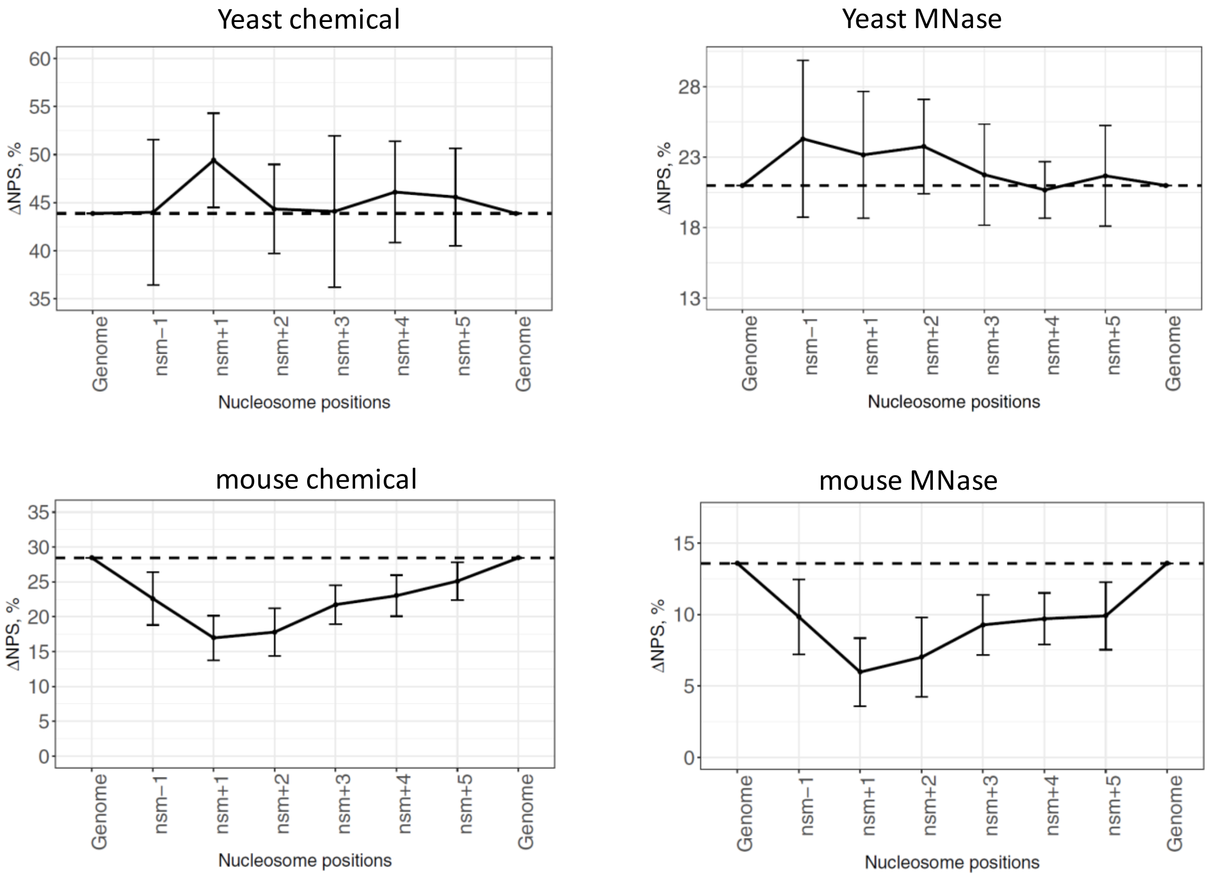

Figure S11. Statistical deviation of ΔNPS values in yeast and mouse chemical and MNase datasets. ΔNPS values for nucleosomes -1 to +5 were calculated by chromosome for each dataset. The average and standard deviation of the ΔNPS values were plotted. The genomic ΔNPS values are denoted by dashed lines.

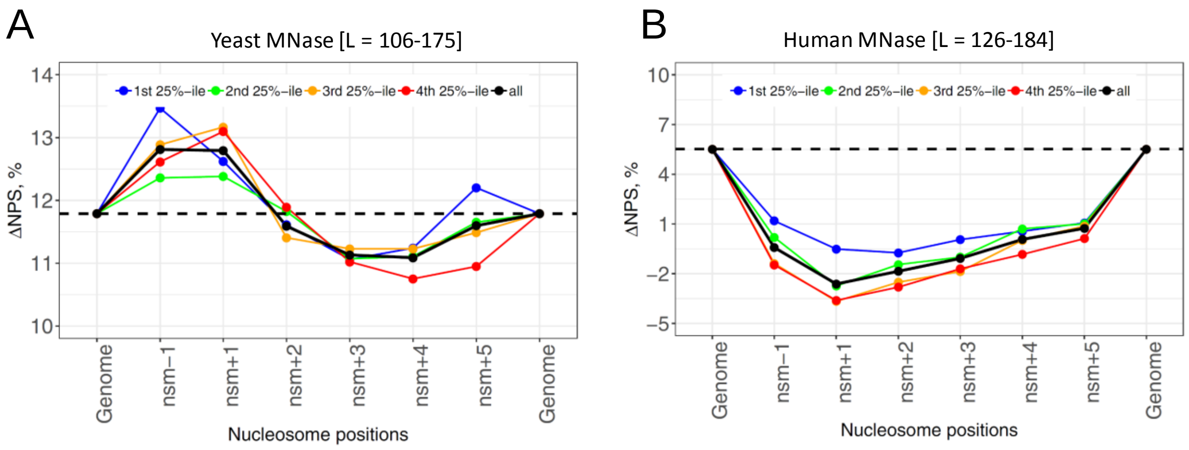

Figure S12. ΔNPS values of nucleosomes -1 to +5 in yeast (A) and human (B) MNase datasets. All paired-end reads (not just 147-bp fragments) in the datasets were used for analysis. The yeast dataset (Cole *et al.* 2011) contains 1,215,378 fragments with the lengths between 106 and 175 bp, whereas the human dataset (Gaffnety *et al*. 2012) contains 2,381,172 fragments with the lengths between 126 and 184 bp. Yeast and human genes are separated into quartiles by transcriptional frequencies. The ΔNPS values of all genes are shown in black. The genomic ΔNPS values are denoted by dashed lines.

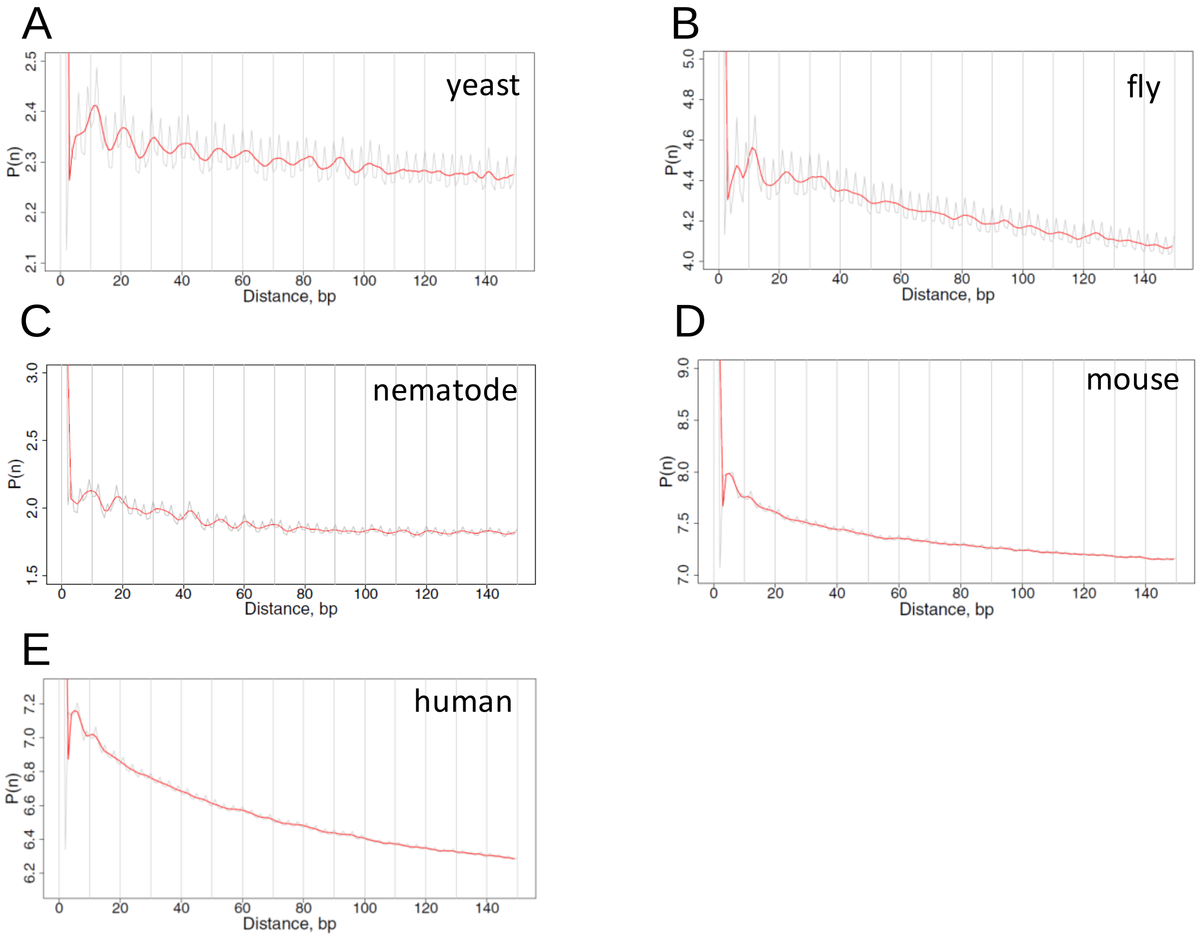

Figure S13. Distance auto-correlation function profiles for SS dinucleotides in yeast (A), fly (B), nematode (C), mouse (D) and human (E) DNA. Genomic fragments [-500 bp, +1000 bp] relative to verified TSSs (position 0) were used for analysis. Both raw (in gray) and 3-bp running average (in red) values were plotted. The distance auto-correlation function follows what was published before (Cui et al. 2012).

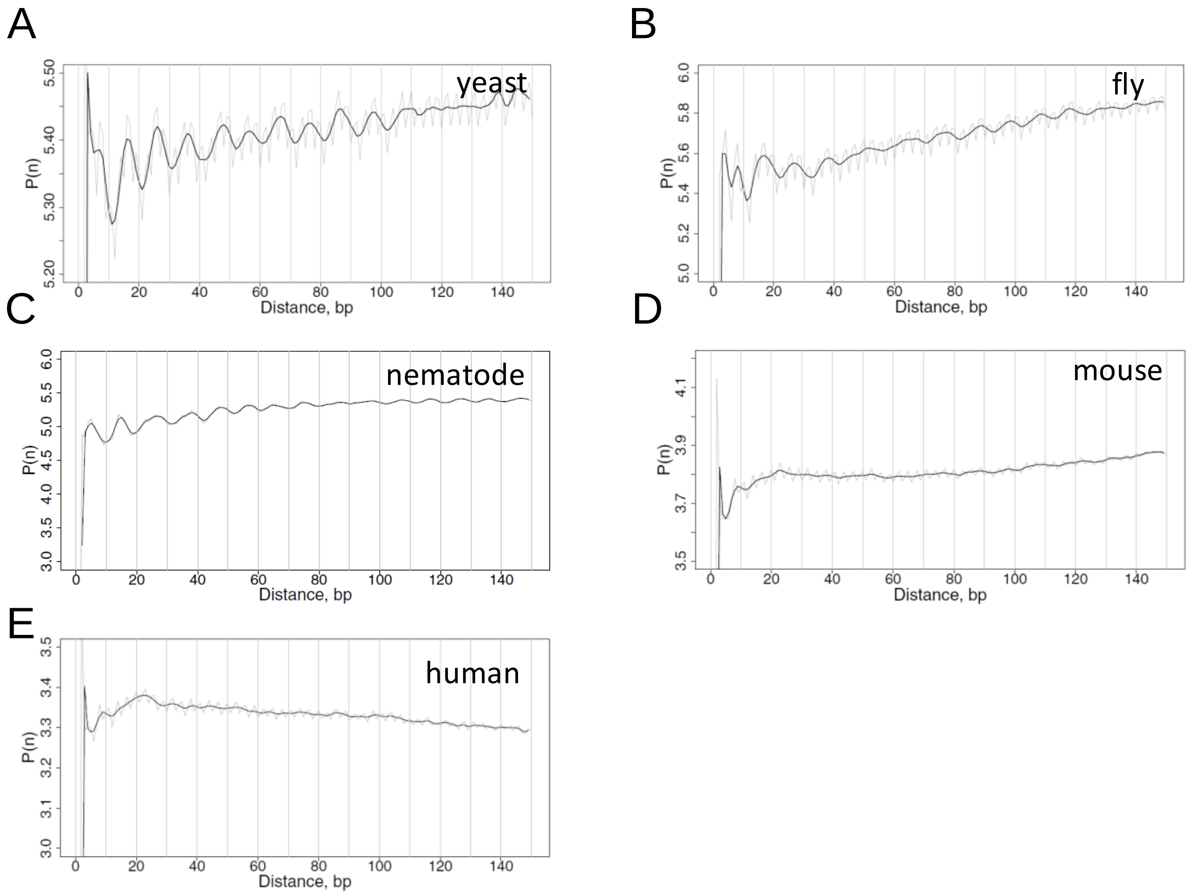

Figure S14. Distance cross-correlation function profiles for WW and SS dinucleotides in yeast (A), fly (B), nematode (C), mouse (D) and human (E) DNA. Genomic fragments [-500 bp, +1000 bp] relative to verified TSSs (position 0) were used for analysis. Both raw (in gray) and 3-bp running average (in black) values were plotted. The distance cross-correlation function follows what was published before (Cui et al. 2012).

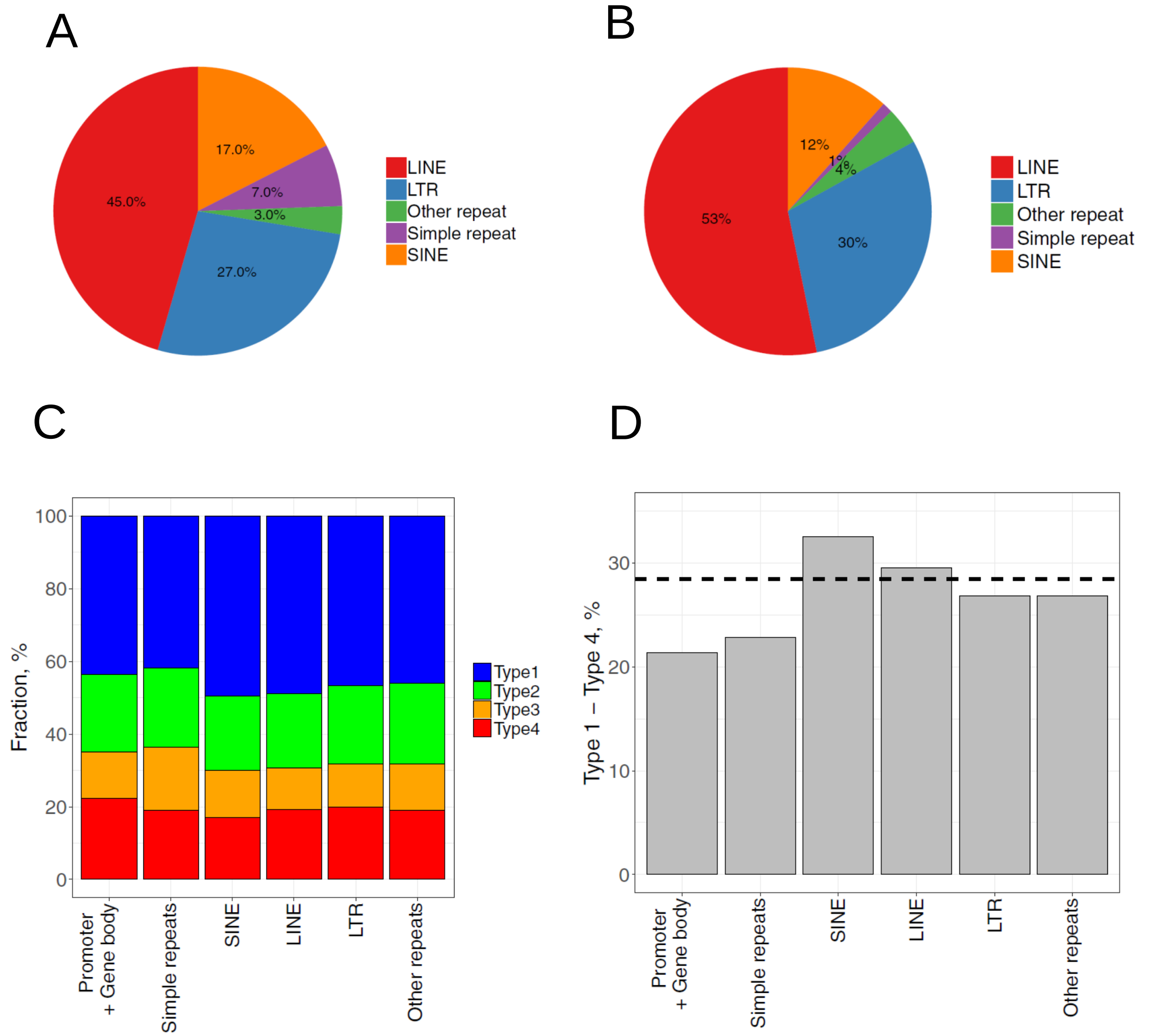

Figure S15. Various nucleosomal DNA sequence patterns in mouse transposable elements (TEs). (A) Fractions of mouse TE families. The fractions of TE families in the mouse genome were taken from literature (http://www.repeatmasker.org/species/mm.html). (B) Fractions of 147-bp mouse nucleosomes residing in TEs grouped by their families. (C) Fractions of 4 types of nucleosomes in TEs grouped by families. (D) Nucleosome ΔNPS values in genic and repetitive DNA regions. The genomic ΔNPS value is indicated by dashed lines.

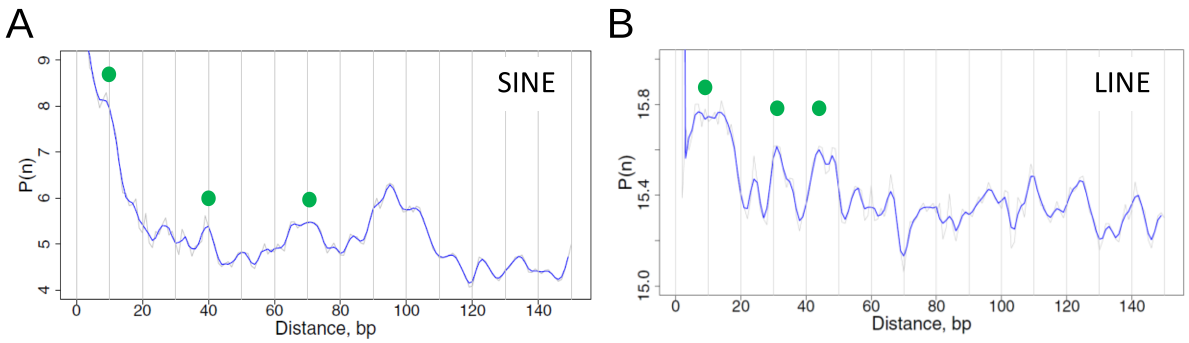

Figure S16. Distance auto-correlation functions for WW dinucleotides in human transposable elements. SINE (A) and LINE (B) elements from human genome (hg18) were used for analysis. Both raw (gray) and 3-bp running averages (blue) of the function values were profiled. Green dots indicate that neighboring WW dinucleotides are separated by a multiple of ~10 bp.

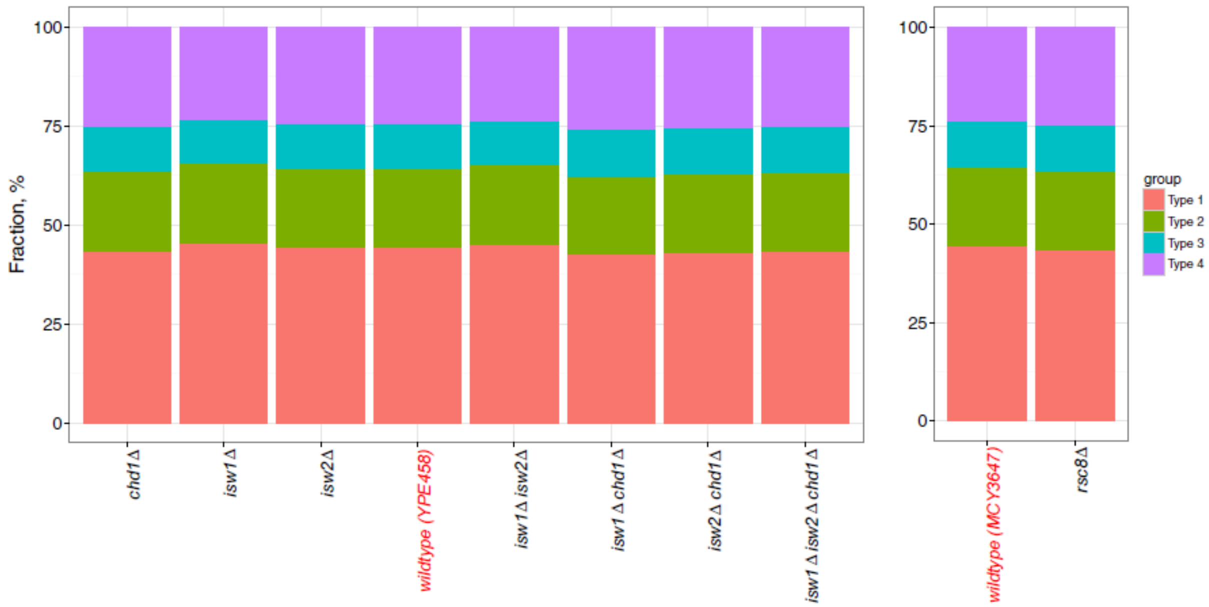

Figure S17. Fractions of 4 types of nucleosomal DNA in yeast wildtype strains and mutants. Notations are the same as in Figure S8A.

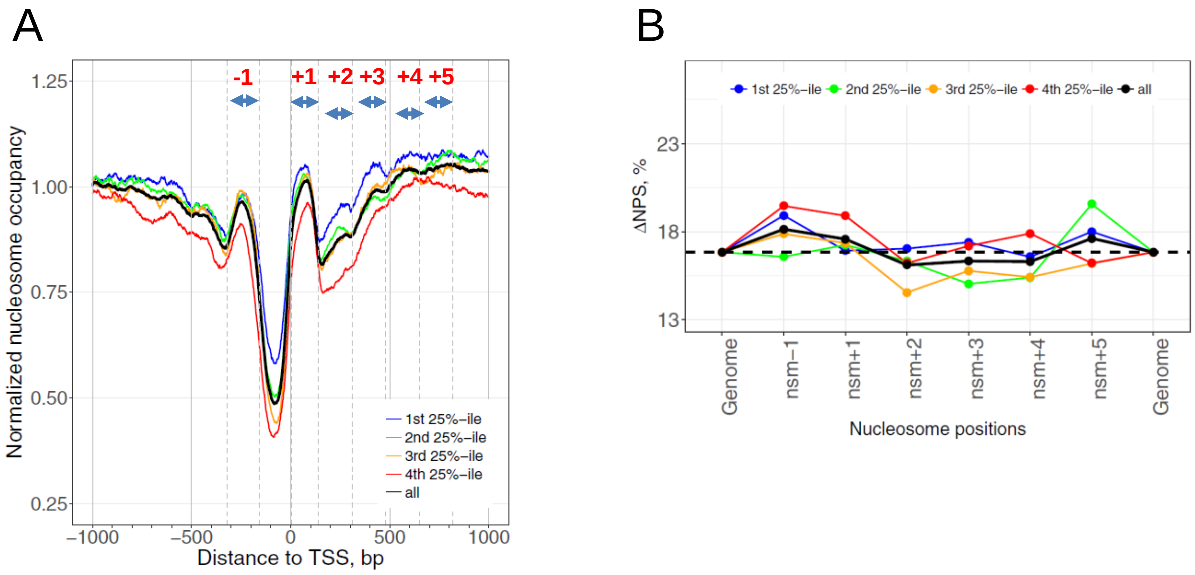

Figure S18. Nucleosome occupancy profiles and ΔNPS values around TSS in a yeast mutant strain (*isw1Δ chd1Δ* double mutant) (Ocampo et al. 2016). (A) Nucleosome occupancy signals ±1 kb of verified TSSs. For the sake of comparison, the ranges of nucleosomes are the same for both the wildtype and mutant strains (Supplementary Table S6). Other notations follow Figure 3. (B) Nucleosome ΔNPS values in genes separated into quartiles by transcriptional frequencies. The ΔNPS values of all genes are shown in black. The genomic ΔNPS values are denoted by dashed lines.

Supplementary References

Brogaard K, Xi L, Wang JP, Widom J. 2012. A map of nucleosome positions in yeast at base-pair resolution. *Nature* **486**: 496-501.

Chereji RV, Kan TW, Grudniewska MK, Romashchenko AV, Berezikov E, Zhimulev IF, Guryev V, Morozov AV, Moshkin YM. 2016. Genome-wide profiling of nucleosome sensitivity and chromatin accessibility in Drosophila melanogaster. *Nucleic Acids Res* **44**: 1036-1051.

Cole HA, Howard BH, Clark DJ. 2011. Activation-induced disruption of nucleosome position clusters on the coding regions of Gcn4-dependent gene extends into neighboring genes. *Nucleic Acids Res* **39**: 9521-9535.

Cui F, Zhurkin VB. 2010. Structure-based analysis of DNA sequence patterns guiding nucleosome positioning in vitro. *J Biomol Struct Dyn* **27**: 821-841.

Cui F, Cole HA, Clark DJ, Zhurkin VB. 2012. Transcriptional activation of yeast genes disrupt intragenic nucleosome phasing. *Nucleic Acids Res* **40**: 10753-10764.

Davey CA, Sargent DF, Luger K, Maeder AW, Richmond TJ. 2002. Solvent mediated interactions in the structure of the nucleosome core particle at 1.9Å resolution. *J Mol Biol* **391**: 1097-1113.

Fuda NJ, Guertin MJ, Sharma S, Danko CG, Martins AL, Siepel A, Lis JT. 2015. GAGA factor maintains nucleosome-free regions and has a role in RNA polymerase II recruitment to promoters. *PLoS Genet.* **11**: e1005108.

Gaffney DJ, McVicker G, Pai AA, Fondufe-Mittendorf YN, Lewellen N, Michelini K, Widom J, Gilad Y, Pritchard JK. 2012. Controls of nucleosome positioning in the human genome. *PLoS Genet* **8**: e1003036.

Ganguli D, Chereji RV, Iben JR, Cole HA, Clark DJ. 2014. RSC-dependent constructive and destructive interference between opposing arrays of phased nucleosomes in yeast. *Genome Res* **24**: 1637-1649.

Ocampo J, Chereji RV, Eriksson PR, Clark DJ. 2016. The ISW1 and CHD1 ATP-dependent chromatin remodelers compete to set nucleosome spacing in vivo. *Nucleic Acids Res* **44**: 4625-4635.

Satchwell SC, Drew HR, Travers AA. 1986. Sequence periodicities in chicken nucleosome core DNA. *J Mol Biol* **191**: 659-675.

Tabuchi TM, Rechtsteiner A, Jeffers TE, Egelhofer TA, Murphy CT, Strome S. 2018. Caenorhabditis elegans sperm carry a histone-based epigenetic memory of both spermatogenesis and oogenesis. *Nat Commun* **9**: 4310.

Voong LN, Xi L, Sebeson AC, Xiong B, Wang JP, Wang X. 2016. Insights into nucleosome organization in mouse embryonic stem cells through chemical mapping. *Cell* **167**: 1555-1570.
